# Dietary conjugated linoleic acid enhances resistance to *Salmonella* infection by promoting PPARγ-mediated metabolic reprogramming and effector function in CD8⁺ T cells

**DOI:** 10.1080/19490976.2026.2657625

**Published:** 2026-04-10

**Authors:** Lei Deng, Xinyu Wang, Dinku Yigezaw Mebratie, Yiting Tang, Jiangang Hu, Jingjing Qi, Mingxing Tian, Yanqing Bao, Lihui Zhu, Shaohui Wang

**Affiliations:** a Shanghai Veterinary Research Institute, Chinese Academy of Agricultural Sciences, Shanghai, People's Republic of China; b Institute of Animal Husbandry and Veterinary Science, Shanghai Academy of Agricultural Sciences, Shanghai, People's Republic of China

**Keywords:** Conjugated linoleic acid, *Salmonella* Typhimurium, CD8^+^ T cells, PPARγ, mucosal immunity

## Abstract

Conjugated linoleic acid (CLA) is a dietary lipid that modulates host–microbiota–immune interactions, yet its mechanistic impact on mucosal defense remains unclear. Here, we show that oral CLA supplementation enhances resistance to *Salmonella* Typhimurium infection and is associated with coordinated changes in gut microbial composition and mucosal immune responses. CLA-enriched commensals, including *Dubosiella* and *Lactobacillus*, were associated with increased production of CLA-derived oxylipins and activation of immune surveillance genes. Functionally, CLA pretreatment reduced *Salmonella* colonization, preserved epithelial integrity, and decreased neutrophilic inflammation without direct antibacterial effects. Single-cell RNA sequencing of ileal intraepithelial lymphocytes revealed that CLA predominantly reprogrammed intestinal CD8⁺ T cells toward an oxidative phenotype and enhanced effector activity. ATAC-seq revealed increased chromatin accessibility at loci associated with metabolic regulation, consistent with transcriptional reprogramming toward oxidative fitness. Mechanistically, CLA directly activated PPARγ signaling to promote mitochondrial biogenesis, oxidative phosphorylation, and the production of IFN-*γ* and granzyme B in CD8⁺ T cells; pharmacologic inhibition of PPARγ attenuated these effects both *in vitro* and *in vivo*. Notably, depletion of CD8⁺ T cells eliminated CLA-mediated protection and abolished early restriction of bacterial dissemination at Peyer’s patches and mesenteric lymph nodes. Although CLA enhanced CD8⁺ T-cell effector programs, antibiotic depletion and fecal microbiota transplantation experiments demonstrated that an intact gut microbiota is necessary for effective protection *in vivo.* Together, these findings identify CLA as a dietary modulator that strengthens mucosal resistance to *Salmonella* by promoting PPARγ-mediated metabolic reprogramming and enhanced effector fitness in intestinal CD8⁺ T cells.

## Introduction

The intestinal mucosa represents one of the most metabolically and immunologically active interfaces between the host and the external environment, integrating dietary inputs with microbial metabolism to shape immune homeostasis and influence susceptibility to inflammation and infection.^1^^,^^2^ *Salmonella enterica* serovar Typhimurium (*S*. Tm) is a major cause of foodborne gastroenteritis and a widely used model for dissecting diet–microbiota–immune interactions during enteric infection.^3-5^ After oral challenge, *S*. Tm preferentially colonizes the distal ileum in mice, a metabolite-rich niche with intense immune surveillance and close host–microbe contact.^6^^,^^7^ *S.* Tm infection disrupts epithelial integrity and triggers robust inflammatory responses,^8^ providing a tractable system to examine how dietary factors shape mucosal immunity and pathogen resistance.

Recent studies suggest that diet–microbiome interactions critically modulate the course and outcome of *Salmonella* infection.^9^^,^^10^ Resident commensals provide colonization resistance by competing for nutrients and ecological niches, producing antimicrobial factors, and maintaining baseline immune tone that limits pathogen expansion.^11^^,^^12^ Dietary perturbations that disrupt microbial communities—such as fiber deprivation or altered fatty-acid intake—promote *S*. Tm expansion and exacerbate intestinal inflammation.^13^ Short-term repetitive exposure to a high-fat diet has been shown to disturb microbial composition and induce a transient state of mucosal and systemic immune suppression, thereby increasing host susceptibility to *S*. Tm infection.^14^ Conversely, propionate-producing *Bacteroides* species restrict *S*. Tm by generating the short-chain fatty acid propionate, which directly inhibits pathogen growth by disrupting intracellular pH homeostasis and protects mice *in vivo*.^15^

Conjugated linoleic acids (CLAs) are a group of positional and geometric isomers of linoleic acid, including *cis*-9, *trans*-11 and *trans*-10, *cis-*12 CLA.^16^^,^^17^ In the gut, CLAs are generated largely through bacterial biohydrogenation of dietary linoleic acid by commensals such as *Lactobacillus*.^17^^,^^18^ CLA supplementation exerts broad immunomodulatory effects, including attenuation of experimental autoimmune encephalomyelitis, amelioration of intestinal inflammation, modulation of macrophage activation, and suppression of tumor progression.^19-22^ Dietary CLA has also been reported to enhance cellular immunity by expanding CD8⁺ lymphocyte populations and increasing CD8⁺-mediated effector functions.^23^ Notably, CLA and its oxidized derivatives can serve as natural ligands for peroxisome proliferator-activated receptor-*γ* (PPARγ), a lipid-sensing nuclear receptor that regulates fatty-acid oxidation, mitochondrial biogenesis, and oxidative phosphorylation.^24^ However, whether CLA-driven PPARγ activation in intestinal mucosal immune cells is required for protection against *S.* Tm infection remains unknown.

In this study, we investigate how dietary CLA influences mucosal defense against *Salmonella* infection. Multi-omics—including 16S rRNA gene sequencing, metabolomics, transcriptomics, single-cell RNA sequencing, and ATAC-seq—revealed that CLA supplementation establishes a metabolically adaptive gut ecosystem enriched in beneficial taxa and immunoregulatory metabolites. Functionally, CLA pretreatment conferred striking protection against enteric infection by limiting pathogen colonization, preserving epithelial integrity, and suppressing inflammatory infiltration. At the cellular level, CLA reprogrammed PPARγ-mediated mitochondrial activation in ileal CD8⁺ T cells, enhancing oxidative phosphorylation and effector molecule production. Epigenomic profiling further revealed widespread chromatin remodeling in intraepithelial lymphocytes, particularly associated with metabolic and immune regulatory genes. Importantly, antibiotic depletion and fecal microbiota transplantation experiments demonstrated that although CLA directly enhances CD8⁺ T-cell effector programs, the presence of an intact gut microbiota is necessary for these immune changes to confer effective mucosal protection *in vivo*.

## Results

### CLA alters gut microbial and mucosal immune landscapes under homeostatic conditions

**Figure 1.**
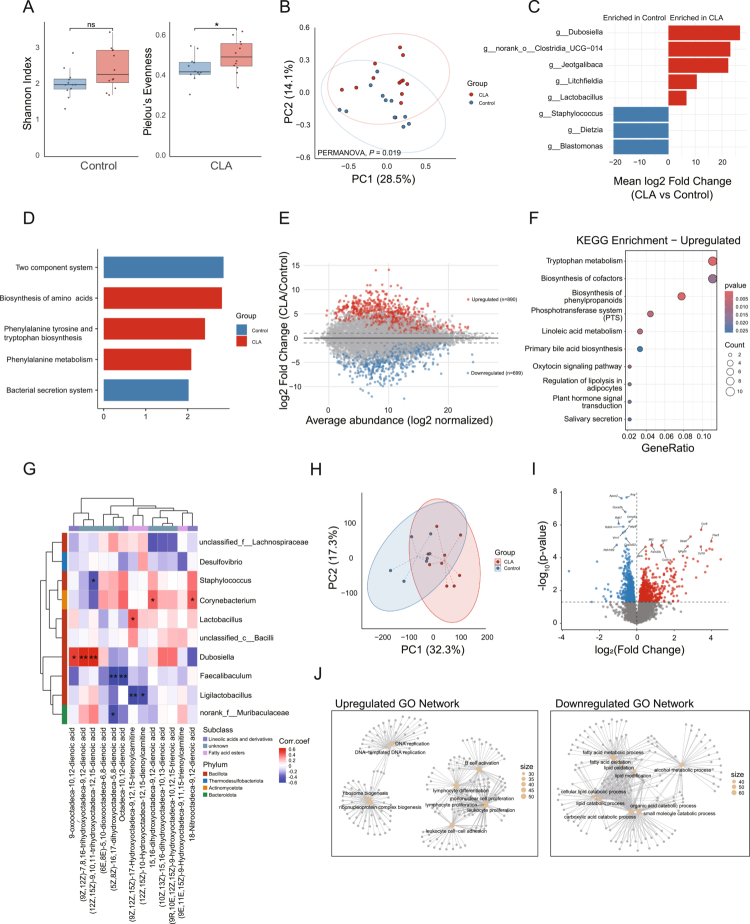
CLA reshapes gut microbial composition and metabolite profiles to establish an immunoregulatory intestinal environment. (A) *α*-diversity of the ileal microbiota in CLA-treated and control mice. (B) PCoA based on Bray–Curtis distances showing distinct microbial community structures between groups. (C) Relative abundance of significantly altered bacterial genera between CLA and control groups. (D) LEfSe analysis of differential KEGG pathways between CLA and control groups. (E) MA plot depicting log_2_ fold changes versus mean metabolite abundance between CLA-treated and control mice. (F) KEGG enrichment analysis of upregulated metabolites in CLA-treated ileal contents. (G) Spearman correlation heatmap illustrating associations between CLA-derived oxylipins and dominant bacterial genera. Red indicates positive correlations, whereas blue indicates negative correlations. Color intensity reflects the strength of the correlation; *p < 0.05, **p < 0.01. (H) Principal component analysis (PCA) of ileal transcriptomic profiles distinguishing CLA-treated and control groups. (I) Volcano plot showing DEGs in the ileum between groups, with the top 10 genes labeled. (J) GO network highlighting enriched biological processes upregulated or downregulated by CLA supplementation. Statistical significance in (A) was determined by unpaired Student’s t-test; *p < 0.05.

To assess how CLA modulates the intestinal ecosystem under steady-state conditions, mice were orally administered CLA or PBS once daily for 14 d. Ileal luminal contents were collected for integrated 16S rRNA gene sequencing, untargeted metabolomics, while ileal tissue samples were harvested for transcriptomic analyzes (Figure S1A). Rarefaction analysis of 16S rRNA gene sequencing data confirmed sufficient depth for downstream analyzes (Figure S1B). CLA supplementation enhanced microbial community evenness (higher Pielou’s index) and tended to increase Shannon diversity, though the latter was not statistically significant (Figure 1A). Principal coordinate analysis (PCoA) revealed a distinct segregation of microbial community structures between CLA-treated and control mice (PERMANOVA, *P* = 0.019; Figure 1B). Differential taxonomic profiling demonstrated that CLA enriched commensal taxa—including *Dubosiella*, *Clostridia UCG-014*, *Jeotgalibaca*, *Litchfieldia*, and *Lactobacillus*—while depleting opportunistic genera such as *Staphylococcus*, *Dietzia*, and *Blastomonas* (Figures 1C and S1C). Functional prediction using Tax4Fun revealed that CLA supplementation enhanced microbial metabolic potential, with increased enrichment in pathways related to biosynthesis of amino acids, phenylalanine tyrosine and tryptophan biosynthesis, and phenylalanine metabolism (Figure 1D). These findings suggest that CLA promotes a functionally reprogrammed microbiota with improved metabolic versatility and energy utilization efficiency.

Untargeted metabolomic profiling of ileal contents revealed extensive remodeling of the luminal metabolite landscape following CLA treatment. Numerous features were differentially abundant, spanning lipid, nucleotide, vitamin, and peptide classes (Figures 1E, S1D,E). KEGG enrichment analysis of upregulated metabolites highlighted tryptophan metabolism and biosynthesis of phenylpropanoids, whereas downregulated metabolites mapped to folate transport and metabolism, and nucleotide metabolism (Figures 1F, S1F). Strikingly, multiple oxidized, hydroxylated, and nitrated CLA-derived species accumulated in CLA-treated mice, indicating *in vivo* transformation of dietary CLA into bioactive oxylipins (Figure S1G). Correlation analysis integrating microbiota and metabolite datasets showed that *Dubosiella* and *Lactobacillus*—both enriched by CLA—positively associated with CLA-derived lipids, whereas *Faecalibaculum*, dominant in controls, exhibited negative correlations (Figure 1G). These results suggest functional cooperation between CLA-responsive commensals and the metabolic conversion of CLA within the gut.

To assess the impact of CLA beyond microbial and metabolic remodeling, we profiled the ileal transcriptome. CLA treatment induced a distinct transcriptional signature, with 849 genes upregulated and 964 downregulated (Figure 1H,I). The volcano plot highlights the top 10 most significantly upregulated and downregulated genes based on fold change and statistical significance (Figure 1I). To provide a global overview of these transcriptional changes, differentially expressed genes were grouped into functional categories by gene ontology (GO) analysis. Upregulated genes were predominantly enriched in immune-stimulatory processes, including lymphocyte differentiation and proliferation (*n* = 97 genes), mononuclear-cell expansion (*n* = 45), and leukocyte adhesion (*n* = 45) (Figure 1J). In contrast, downregulated genes were mainly associated with fatty acid metabolic process (*n* = 69) and small molecule catabolic process (*n* = 60) (Figure 1J).

Collectively, these findings reveal that oral CLA supplementation reprograms the gut ecosystem at multiple levels—microbial composition, metabolic output, and mucosal immune tone—thereby establishing a metabolically adaptive and immunologically poised intestinal environment that may enhance host resilience to subsequent inflammatory or infectious challenges.

### CLA pretreatment enhances mucosal resistance to Salmonella infection

**Figure 2.**
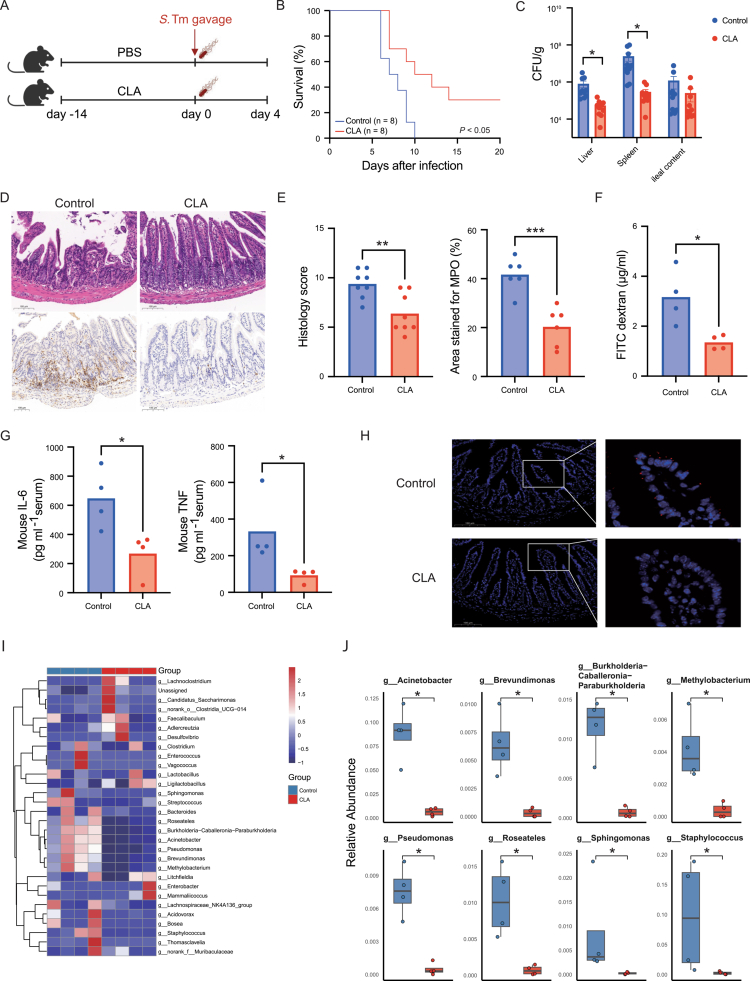
CLA supplementation protects against *Salmonella* infection by preserving mucosal integrity and reducing inflammation. (A) Experimental design. (B) Kaplan–Meier survival curves (*n* = 8 per group). (C) Bacterial loads in liver, spleen, and ileal contents from infected mice. (D) Representative histology and MPO immunostaining of ileal tissues. (E) Quantification of histological inflammation and MPO-positive cells. (F) Plasma FITC–dextran concentrations between CLA and control groups. (G) Serum proinflammatory cytokine levels (IL-6 and TNF) measured by ELISA. (H) FISH images showing *S.* Tm (red) and host cell nuclei stained with DAPI (blue) in ileal sections. Scale bars, 100 μm. (I) Heatmap of the top 30 differentially abundant genera in the ileum (CLA vs control group). (J) Relative abundance of key bacterial genera significantly altered by CLA treatment. Data are presented as mean ± SEM; Statistical significance was determined by unpaired Student’s t-test for (C, E, F, G)) and Wilcoxon’s test for J; *p < 0.05, **p < 0.01, ***p < 0.001. Results are representative of at least two independent experiments.

To determine whether CLA supplementation confers protection against enteric bacterial infection, mice were pre-treated with CLA by daily oral gavage for 14 d before challenge with *S.* Tm (Figure 2A). CLA-treated mice exhibited markedly improved survival compared with PBS-treated controls (Figure 2B) and showed significantly lower bacterial burdens in both liver and spleen (Figure 2C). Histopathological examination revealed extensive epithelial erosion, crypt hyperplasia, and dense inflammatory-cell infiltration in control mice, whereas CLA-treated animals maintained well-preserved mucosal architecture and displayed substantially reduced histological inflammation scores (Figure 2D,E). Immunohistochemical staining for myeloperoxidase (MPO), a marker of neutrophilic inflammation, confirmed reduced MPO-positive areas in CLA-treated intestines (Figure 2D,E). Intestinal barrier integrity assessed by oral FITC–dextran administration demonstrated lower serum fluorescence intensity in CLA-treated mice, indicating improved epithelial permeability (Figure 2F). Consistently, circulating levels of the pro-inflammatory cytokines TNF and IL-6 were significantly decreased in the CLA group (Figure 2G). Fluorescence in situ hybridization (FISH) for *S.* Tm further revealed abundant bacterial adherence and epithelial invasion in control mice but minimal signal in CLA-treated counterparts (Figure 2H).

To dissect the contribution of the gut microbiota to this protective phenotype, we next profiled microbial communities following infection. Although *α*-diversity indices remained comparable between groups (Figure S2A,B), *β*-diversity analysis showed clear segregation of microbial communities (Figure S2C). CLA pretreatment preserved microbiota stability after *S.* Tm challenge, whereas control mice displayed pronounced dysbiosis (Figure 2I,J). Inflammation-associated taxa—including *Acinetobacter*, *Brevundimonas*, *Burkholderia-Caballeronia-Paraburkholderia*, *Pseudomonas*, *Roseateles*, *Sphingomonas*, and *Staphylococcus*—were markedly expanded in infected controls but remained low in CLA-treated animals (Figures 2I,J and S2D). Functional inference indicated that CLA maintained microbial pathways related to phosphotransferase system, ABC transporters, and general metabolism (Figure S2E). To rule out a direct antibacterial effect, *S.* Tm was cultured *in vitro* with escalating CLA concentrations (25–200 µM); no significant inhibition of bacterial growth was observed (Figure S2F).

Collectively, these findings demonstrate that dietary CLA supplementation enhances host resistance to *S.* Tm infection by restricting pathogen colonization, preserving epithelial integrity, and dampening inflammatory responses. This protection is not due to direct antibacterial activity but rather to CLA-mediated stabilization of the gut microbiota and strengthening of mucosal barrier function.

### CLA promotes metabolic activation of intestinal CD8⁺ T cells

**Figure 3.**
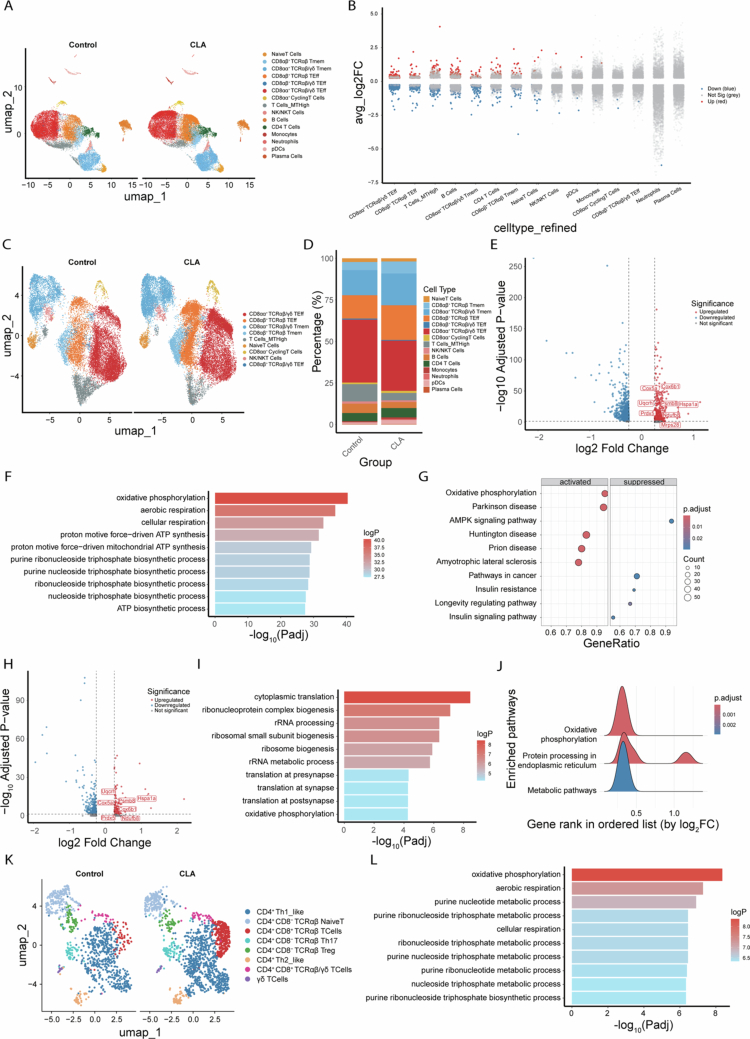
CLA reprograms the intestinal immune landscape and metabolic states of CD8⁺ T cells during *Salmonella* infection. (A) UMAP visualization of ileal CD45⁺ IELs from CLA-treated and control mice (*n* = 3 per group). (B) Global transcriptional changes across major immune cell subsets. (C) Reclustering of CD8⁺ T cells revealing distinct subpopulations. (D) Relative proportions of major immune cell populations in CLA-treated and control mice. (E) Volcano plot of DEGs in CD8αα⁺ TCRαβ/γδ effector T cells. (F) GO enrichment of upregulated genes in CD8αα⁺ TCRαβ/γδ effector T cells from CLA-treated mice. (G) KEGG pathway enrichment analysis. (H) Volcano plot of DEGs in CD8αβ⁺ TCRαβ effector T cells. (I) GO enrichment of upregulated genes in CD8αβ⁺ TCRαβ effector T cells from CLA-treated mice. (J) GSEA ridge plot showing positive enrichment of oxidative phosphorylation–related pathways in CLA-treated CD8αβ⁺ TCRαβ effector T cells. (K) Reclustering of CD4⁺ T cells revealing distinct subpopulations. (L) GO enrichment of upregulated genes in CD4⁺CD8αα⁺ T cell subsets.

To assess whether CLA modulates mucosal immune responses during *Salmonella* infection, intraepithelial lymphocytes (IELs) were isolated from the ileal epithelium of *Salmonella*-infected CLA- and PBS-treated mice, and CD45⁺ cells were sorted for single-cell RNA sequencing (scRNA-seq). Unsupervised clustering identified fifteen major immune populations, including naïve and effector CD8⁺ T cells, CD4⁺ T cells, B cells, NK/NKT cells, monocytes, neutrophils, pDCs, and plasma cells (Figure 3A). Comparative transcriptional profiling revealed that both CD8αα⁺TCRαβ⁺/γδ⁺ and CD8αβ⁺TCRαβ⁺ effector T cells exhibited the most pronounced transcriptomic alterations following CLA supplementation (Figure 3B). Given that CD8⁺ T cells represent the dominant IEL subset, we performed focused reclustering of CD8⁺ T cells, resolving distinct subsets corresponding to memory-like, effector, and cycling states (Figure 3C). Among these, CD8αβ⁺TCRαβ⁺ effector T cells (defined by *Cd8b1*, *Trac*, and *Gzmb* expression) were expanded in CLA-treated mice, whereas CD8αα⁺TCRαβ⁺/γδ⁺ effector T cells (defined by *Trac*, *Trdc*, and *Gzmb*) predominated in controls (Figures 3D and S3A,B). Differential expression analysis revealed that CLA induced robust upregulation of mitochondrial and metabolic gene programs in CD8αα⁺TCRαβ⁺/γδ⁺ effector T cells. Upregulated genes included mitochondrial ribosomal and electron transport chain components—*Mrps28*, *Ndufb8*, *Cox5a*, *Cox6b1*, and *Uqcrh*—along with antioxidant and stress-response genes such as *Prdx5*, *Psmb8*, and *Hspa1a*, collectively indicating enhanced mitochondrial biogenesis, oxidative phosphorylation, and redox homeostasis (Figure 3E). GO enrichment analyzes consistently highlighted mitochondrial oxidative and energy-generating pathways, such as oxidative phosphorylation, aerobic respiration, and ATP biosynthesis (Figure 3F). KEGG pathway mapping also revealed activation of oxidative phosphorylation along with downregulation of AMPK signaling, suggesting a metabolic shift toward efficient mitochondrial energy utilization and improved metabolic fitness (Figure 3G). Similar upregulation of oxidative phosphorylation–related genes was also observed in CD8αβ⁺TCRαβ⁺ effector T cells, and GO and KEGG showed enrichment of oxidative phosphorylation and protein processing in endoplasmic reticulum, confirming that CLA broadly enhances CD8⁺ T-cell metabolic activity (Figure 3H–J).

Furthermore, given the immunoregulatory relevance of CD4⁺CD8αα⁺ intraepithelial T cells in maintaining mucosal homeostasis,^25^ we next profiled CD4⁺ IEL populations at higher resolution. Subclustering delineated eight transcriptionally distinct populations, including Th1-like, Th2-like, Th17, regulatory (Treg), and CD4⁺CD8αα⁺ effector-like T cells (Figure 3K). The CD4⁺CD8αα⁺ subset, characterized by *Cd8a* and *Gzma* expression, was significantly increased in CLA-treated mice, suggesting that CLA also promotes the expansion of dual-lineage effector populations (Figure S3C,D). GO analysis further revealed enrichment of oxidative phosphorylation–related pathways, indicating enhanced metabolic activation within this subset (Figure 3L).

To delineate functional differences among key IEL subsets, we compared effector and metabolic gene expression across CD4⁺CD8αα⁺ T cells, CD8αα⁺TCRαβ⁺/γδ⁺ effector T cells, and CD8αβ⁺TCRαβ⁺ effector T cells (Figure S4). In CD4⁺CD8αα⁺ T cells, CLA predominantly induced the immunoregulatory gene *Il10*, with modest increases in mitochondrial and fatty acid oxidation–related genes (*Cpt1b, Mrpl47,* and *Mrps28*). In contrast, CD8αα⁺TCRαβ⁺/γδ⁺ effector T cells showed strong upregulation of cytotoxic genes (*Gzma, Gzmb,* and *Nkg7*) together with activation of mitochondrial oxidative phosphorylation pathways, indicative of an enhanced innate-like cytotoxic phenotype. Notably, CD8αβ⁺TCRαβ⁺ effector T cells exhibited coordinated induction of mitochondrial metabolic genes alongside increased expression of adaptive effector molecules (*Nkg7* and *Ifng*). Given the essential role of IFN-*γ* in controlling *Salmonella* infection,^26^^,^^27^ these features suggest that CD8αβ⁺TCRαβ⁺ effector T cells may represent a key metabolically reprogrammed effector population contributing to CLA-mediated protection.

Collectively, these results demonstrate that CLA supplementation drives a coordinated upregulation of mitochondrial oxidative phosphorylation in intestinal CD8⁺ T cells, thereby enhancing their metabolic competence and effector potential during enteric infection.

### CLA reshapes intercellular communication networks and attenuates inflammatory signaling

**Figure 4.**
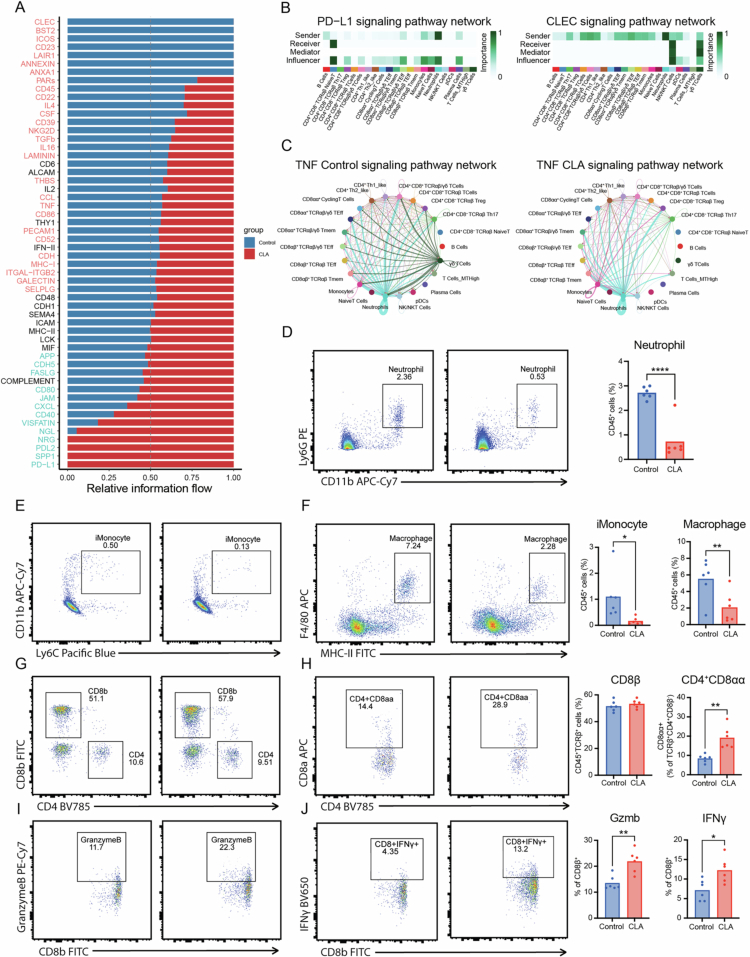
CLA reshapes intercellular communication and effector function of intestinal T cells during *Salmonella* infection. (A) Information flow of signaling pathways in control and CLA-treated groups analyzed by CellChat. (B) CellChat analysis of PD-L1 and CLEC signaling pathway networks among ileal immune cell subsets. (C) TNF signaling pathway networks in control (left) and CLA-treated (right) mice visualized by CellChat. (D) Representative flow cytometry plots and proportion of neutrophils (CD11b⁺Ly6G⁺). (E–F) Representative flow cytometry plots and proportion of inflammatory monocytes (CD11b⁺Ly6C⁺) and macrophages (F4/80⁺MHC-II⁺). (G–H) Representative flow cytometry plots and proportion of CD8β⁺, CD4⁺, and CD4⁺CD8αα⁺ T cells. (I–J) Representative intracellular staining and proportion of granzyme B⁺ and IFN-γ⁺ CD8⁺ T cells. Data are presented as mean ± SEM; Statistical significance was determined by unpaired Student’s t-test for (D-J); *p < 0.05, **p < 0.01, ****p < 0.0001. Results (D-J) are representative of at least two independent experiments.

To delineate how CLA modulates intercellular communication within the intestinal immune compartment, we applied CellChat analysis to the single-cell transcriptomic atlas of ileal immune cells.^28^ CLA supplementation profoundly remodeled the global ligand–receptor signaling landscape, reducing both the overall number and strength of inferred intercellular interactions (Figure S5A,B). This contraction of communication networks corresponded to a shift from pro-inflammatory to immunoregulatory signaling circuits. We further assessed the information flow for each signaling pathway, determined by the combined communication probabilities between all pairs in the inferred network.^28^^,^^29^ Comparison of the information flow between groups revealed a marked reduction in activity in pathways such as CLEC, BST2, ICOS, CD23, LAIR1, and ANXA1, all of which are associated with antigen recognition, costimulation, and leukocyte activation in CLA-treated mice. In contrast, pathways including NRG, PD-L2, SPP1, and PD-L1 were enhanced, reflecting an enrichment of tolerogenic and inhibitory communication axes (Figure 4A). Within the PD-L1 pathway, neutrophils emerged as dominant “sender” and “influencer” cells, whereas T cells showed enhanced “receiver” activity, suggesting that CLA strengthens inhibitory crosstalk from myeloid cells to T cells and other lymphoid populations (Figure 4B). Conversely, the CLEC signaling network—primarily driven by neutrophils and NK/NKT cells in control mice—was markedly attenuated after CLA treatment, implying reprogramming of myeloid–T cell interactions toward a less inflammatory state (Figure 4B). Interestingly, regarding TNF signaling pathway, control mice exhibited a dense, highly interconnected TNF network among multiple immune subsets, with prominent interactions between neutrophils, Th17 cells, and effector CD8⁺ T cells (Figure 4C). This configuration reflects an active pro-inflammatory microenvironment characterized by TNF-driven amplification loops. In contrast, CLA treatment markedly reduced both the intensity and connectivity of TNF-mediated interactions, indicating that CLA suppresses excessive TNF-driven immune activation and dampens intercellular inflammatory propagation (Figure 4C).

We further validated immune cell population changes by flow cytometry (Figure S5C,D). Although no robust changes in neutrophils or inflammatory monocytes were detected in the single-cell RNA-seq dataset (Figure 3D), likely due to their low abundance within the CD45⁺ IEL compartment, flow cytometric analysis revealed a significant reduction in neutrophils, inflammatory monocytes, and macrophages following CLA treatment (Figure 4D–F). These differences likely reflect methodological and compartment-specific sensitivities between the two approaches, with flow cytometry providing a more quantitative assessment of low-frequency inflammatory myeloid populations at the epithelial interface, and scRNA-seq offering complementary transcriptomic characterization. Across both datasets, CD8⁺ T cells remained the dominant IEL population, and the proportion of CD4⁺CD8αα⁺ intraepithelial T cells was elevated following CLA treatment (Figure 4G,H). Interestingly, effector molecules such as IFN-*γ* and granzyme B were increased in CD8⁺ T cells, reflecting enhanced cytotoxic and regulatory competence (Figure 4I,J). Together, these results demonstrate that CLA supplementation restrains pro-inflammatory cytokine cascades while reinforcing inhibitory signaling pathways and cytotoxic T-cell functionality.

### CLA remodels the chromatin landscape to lock in effector–metabolic states

**Figure 5.**
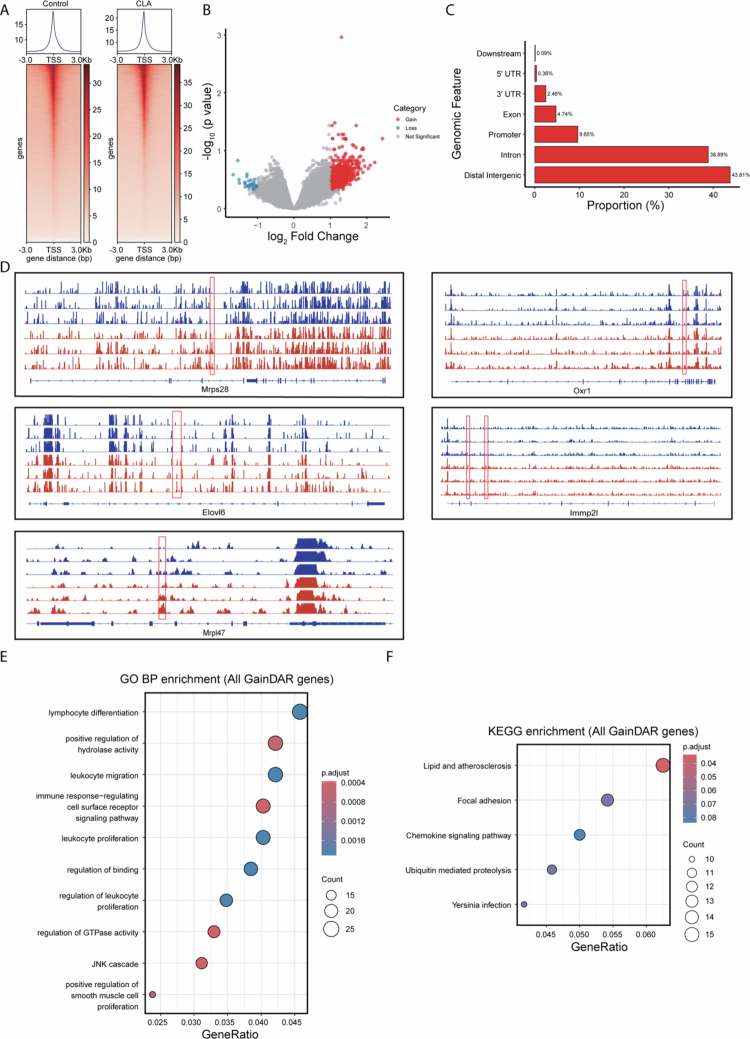
CLA promotes chromatin accessibility and transcriptional activation of mitochondrial and metabolic genes in immune cells during *Salmonella* infection. (A) Heatmaps and average profiles of ATAC-seq signal intensity around transcription start sites (TSS; ± 3 kb) in control and CLA-treated groups. (B) Volcano plot showing differentially DARs between CLA-treated and control CD45⁺ T cells. (C) Genomic distribution of gain DARs. (D) Representative IGV browser tracks for selected genes (*Mrps28*, *Elovl6*, *Mrpl47*, *Oxr1*, and *Immp2l*). Significantly altered peaks are outlined in red; blue and red tracks indicate control and CLA groups, respectively. (E) GO enrichment analysis of genes associated with gain DARs. (F) KEGG pathway enrichment of gain DAR-associated genes.

To determine whether CLA influences epigenetic regulation of mucosal immune cells during infection, we performed ATAC-seq on CD45⁺ IELs isolated from the ileal of *S.* Tm infected mice treated with CLA or PBS. Global accessibility profiles revealed a marked increase in open chromatin regions following CLA treatment (Figure 5A). CLA supplementation resulted in a distinct chromatin accessibility profile, with 1099 regions showing increased and 46 regions showing decreased accessibility relative to controls (Figure 5B). Annotation of differential peaks indicated that most Gain DARs were located in distal intergenic and intronic regions, consistent with the activation of transcriptional enhancer elements (Figure 5C). Representative genome tracks demonstrated enhanced accessibility at metabolic genes such as *Mrps28*, *Elovl6*, *Mrpl47*, *Oxr1*, and *Immp2l*, which encode proteins involved in mitochondrial ribosomal assembly, fatty acid elongation, oxidative stress defense, and mitochondrial protein import, consistent with enhanced transcriptional programs supporting oxidative metabolism (Figure 5D). Functional enrichment analysis of genes associated with Gain DARs demonstrated significant enrichment of biological processes linked to lymphocyte differentiation, positive regulation of hydrolase activity, and leukocyte migration/proliferation (Figure 5E). KEGG pathway analysis further identified lipid and atherosclerosis as prominent category (Figure 5F). Together, these data indicate that CLA exposure promotes widespread chromatin opening in intraepithelial lymphocytes, particularly at loci associated with metabolic and immunoregulatory genes.

### CLA enhances mitochondrial metabolism and effector function of CD8⁺ T cells through PPARγ activation

**Figure 6.**
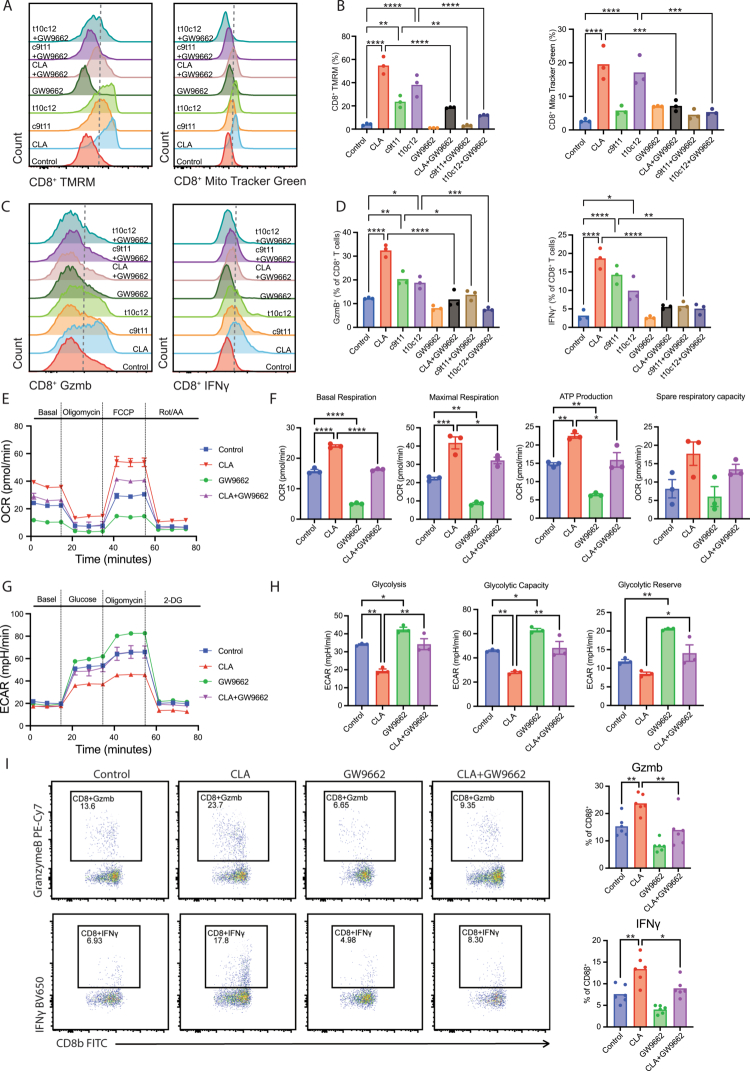
CLA enhances mitochondrial metabolism and effector function of CD8⁺ T cells through PPARγ activation. (A, B) Representative flow cytometry plots and proportion of CD8⁺ T cells positive for TMRM and MitoTracker Red fluorescence after treatment with vehicle, CLA (mixture of c9t11 and t10c12), c9t11, t10c12, or the PPARγ inhibitor GW9662. (C, D) Representative flow cytometry plots and proportion of granzyme B⁺ and IFN-γ⁺ CD8⁺ T cells following treatment with vehicle, CLA (mixture of c9t11 and t10c12), c9t11, t10c12, or GW9662. (E, F) Seahorse extracellular flux analysis showing OCR of CD8⁺ T cells treated with vehicle, CLA (mixture of c9t11 and t10c12), or GW9662. (G, H) Seahorse analysis showing ECAR of CD8⁺ T cells under the same treatment conditions. (I) Representative flow cytometry plots and quantification of granzyme B⁺ and IFN-γ⁺ CD8⁺ T cells isolated from ileal intraepithelial lymphocytes under the indicated treatment conditions. c9t11 (*cis*-9, *trans*-11 CLA), t10c12 (*trans*-10, *cis*-12 CLA). Data are presented as mean ± SEM; Statistical significance was determined by One-way ANOVA test (B, D, F, H, I); *p < 0.05, **p < 0.01, ***p < 0.001, ****p < 0.0001. Results are representative of at least two independent experiments.

Given that CLA is a known endogenous ligand of PPARγ,^24^ we next examined whether CLA regulates CD8⁺ T cells mitochondrial metabolism via PPARγ signaling. Purified CD8⁺ T cells were treated *in vitro* with increasing concentrations of CLA (0–100 µM). All concentrations maintained >95% cell viability, and 50 µM CLA induced a significant enhancement in mitochondrial mass and membrane potential, as measured by MitoTracker Green and TMRM staining (Figure S6A). This concentration (50 µM) was selected for subsequent assays due to its optimal efficacy and biological relevance, as it falls within the physiologically relevant range of 10–70 µM CLA in human sera^30^ and aligns with previous studies demonstrating its immune-modulatory effects on immune cells.^21^^,^^31^^,^^32^ To assess PPARγ dependence, cells were co-treated with GW9662, a selective PPARγ antagonist.^33^ CLA treatment—either as individual isomers or as a mixture—markedly increased mitochondrial mass and membrane potential, whereas co-treatment with GW9662 abrogated these effects (Figure 6A,B). At the functional level, CLA significantly increased the production of IFN-*γ* and granzyme B in CD8⁺ T cells, while PPARγ inhibition suppressed these effector responses (Figure 6C,D). Quantitative PCR analysis confirmed that CLA upregulated key mitochondrial and metabolic genes—including *Cpt1b*, *Mrps28*, and *Mrpl47*—which are involved in fatty-acid oxidation and mitochondrial translation; co-treatment with GW9662 largely reversed these transcriptional effects (Figure S6B).

To determine whether CLA-induced metabolic remodeling depends on PPARγ activation *in vivo* and whether PPARγ is essential for the protective effects of CLA against *Salmonella* infection, we performed *ex vivo* metabolic profiling of flow-sorted CD8⁺ T cells purified from ileal IELs of CLA- and PBS-treated mice, together with complementary inhibition experiments (Figure S6C). Seahorse extracellular flux analysis revealed that CLA markedly increased both basal, maximal oxygen-consumption rates (OCR), and ATP production, indicating enhanced mitochondrial oxidative phosphorylation capacity (Figure 6E,F). In contrast, extracellular acidification rate (ECAR) analysis showed reduced glycolysis, glycolytic capacity, and glycolytic reserve, reflecting a metabolic shift toward oxidative metabolism (Figure 6G,H). Notably, pharmacologic inhibition of PPARγ significantly attenuated the CLA-induced increase in IFN-*γ* and granzyme B production in CD8⁺ T cells isolated from ileal IELs (Figure 6I), indicating that PPARγ signaling contributes to CLA-associated effector reprogramming in this compartment. Consistently, histopathological analysis revealed exacerbated mucosal inflammation and increased bacterial burdens in GW9662-treated mice, further confirming that PPARγ activation is essential for CLA-mediated metabolic and protective effects (Figure S6D–F). Importantly, we further examined intestinal PPARγ expression under different conditions. Consistent with previous reports,^34^ *S.* Tm infection reduced intestinal PPARγ expression, whereas CLA supplementation restored PPARγ levels during infection (Figure S6G).

Collectively, these findings demonstrate that CLA activates a PPARγ-mediated metabolic program that enhances mitochondrial biogenesis, oxidative capacity, and effector function of CD8⁺ T cells, thereby mediating the protective immunity against *Salmonella* infection.

### CD8⁺ T cells are required for CLA-mediated protection and early restriction of Salmonella invasion

**Figure 7.**
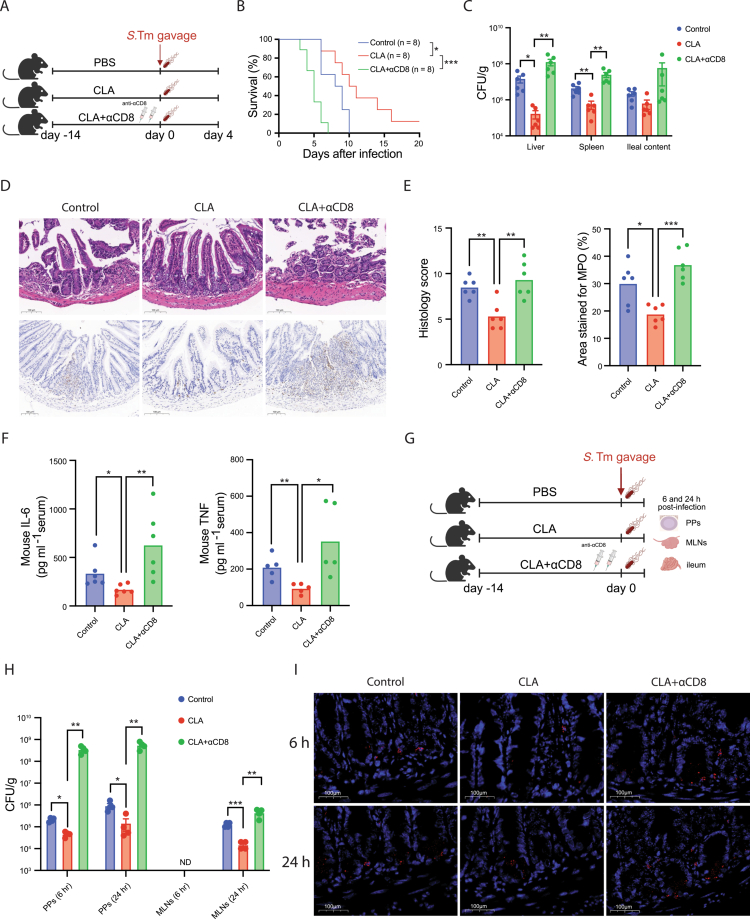
CD8⁺ T cells contribute to CLA-associated mucosal protection during *Salmonella* infection. (A) Experimental design. (B) Kaplan–Meier survival curves (*n* = 8 per group). (C) Bacterial loads in liver, spleen, and ileal contents. (D, E) Representative H&E and MPO-stained ileal sections and quantification of histological inflammation scores and MPO⁺ areas. (F) Serum TNF and IL-6 levels measured by ELISA. (G) Experimental design. (H) *Salmonella* CFU in Peyer’s patches (PPs) and mesenteric lymph nodes (MLNs) at 6 h and 24 h post-infection (*n* = 4 per group). No bacteria were detected in MLNs at 6 h post-infection (ND, not detected). (I) FISH analysis of *Salmonella* localization in the intestinal epithelium of CLA-treated mice with or without CD8⁺ T-cell depletion. Data are shown as mean ± SEM; Statistical significance was determined by unpaired Student’s t-test for (C, E, F, H); *p < 0.05, **p < 0.01, ***p < 0.001, ****p < 0.0001. a representative of at least two experiments.

To determine whether CD8⁺ T cells contribute to the protective effects associated with CLA during *S. Tm* infection, we depleted CD8⁺ T cells using an anti-CD8α monoclonal antibody prior to infection (Figure 7A). Efficient depletion of CD8⁺ T cells in both the intestinal mucosa and peripheral lymphoid organs was confirmed by flow cytometry (Figure S7A,B). Consistent with our previous observations, CLA-treated mice displayed significantly improved survival and markedly lower bacterial burdens in the liver and spleen compared with controls (Figure 7B,C). However, depletion of CD8⁺ T cells markedly attenuated these CLA-associated protective effects (Figure 7B,C). CLA-treated mice subjected to CD8⁺ T-cell depletion exhibited significant higher bacterial loads and more severe disease compared with non-depleted CLA-treated controls (Figure 7D,E). Furthermore, CD8-depleted mice showed pronounced disruption of the intestinal barrier accompanied by elevated levels of proinflammatory cytokines TNF and IL-6 (Figure 7F). These results demonstrate that CD8⁺ T cells are required for the full protective phenotype observed under CLA supplementation.

Given that *S.* Tm infection proceeds through well-defined anatomical checkpoints—initial invasion of Peyer’s patches (PPs), expansion in mesenteric lymph nodes (MLNs), and subsequent systemic dissemination—we examined whether CLA targets early PP invasion and whether this effect depends on CD8⁺ T cells (Figure 7G). At 6 and 24 h post infection, CLA-treated mice exhibited significantly reduced bacterial burdens in both PPs and MLNs, whereas this early restriction was completely abolished following CD8⁺ T-cell depletion (Figure 7H), suggesting that CD8⁺ T cells contribute to CLA-associated early containment of bacterial dissemination. Furthermore, FISH revealed that CLA markedly limited *Salmonella* translocation across the intestinal epithelium, an effect that was lost upon CD8⁺ T-cell depletion (Figures 7I, S7C). Together, these results support a critical role for CD8⁺ T cells in mediating the early containment of intestinal *Salmonella* infection associated with CLA supplementation.

### The intestinal microbiota is required for CLA-associated protection against Salmonella infection

**Figure 8.**
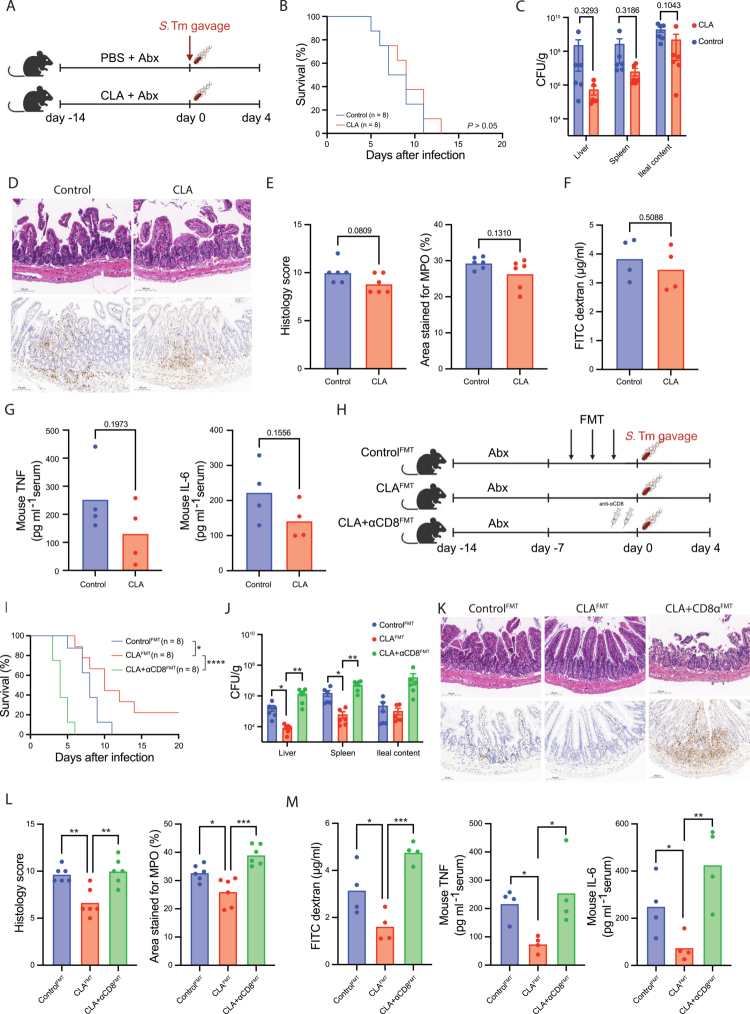
An intact gut microbiota is required for CLA-associated protection against *Salmonella* infection. (A) Experimental design of antibiotic depletion experiments. (B) Kaplan–Meier survival curves of mice after *S.* Tm infection (*n* = 8 per group). (C) Bacterial loads in liver, spleen, and ileal contents. (D) Representative H&E and MPO-stained ileal sections. (E) Quantification of histological inflammation scores and MPO⁺ areas. (F) Plasma FITC–dextran concentration in control and CLA-treated mice. (G) Serum TNF and IL-6 levels. (H) Experimental design of fecal microbiota transplantation from CLA-treated or control donors into antibiotic-pretreated recipients. (I) Kaplan–Meier survival curves of FMT recipient mice after *S.* Tm infection (*n* = 8 per group). (J) Bacterial loads in liver, spleen, and ileal contents of FMT recipients. (K) Representative H&E and MPO-stained ileal sections of FMT recipients. (L) Quantification of histological inflammation scores and MPO⁺ areas in CLA-FMT recipients. (M) Plasma FITC–dextran, TNF, and IL-6 levels in CLA-conditioned microbiota recipients. Data are presented as mean ± SEM; Statistical significance was determined by unpaired Student’s t-test for (C, E, F, G, J), and One-way ANOVA test for (L, M); *p < 0.05, **p < 0.01, ***p < 0.001, ****p < 0.0001. Results are representative of at least two independent experiments.

To assess the contribution of the intestinal microbiota to the *in vivo* protective effects of CLA, mice were pretreated with a broad-spectrum antibiotic cocktail prior to CLA supplementation and *S.* Tm challenge (Figure 8A). As expected, antibiotic depletion largely abolished the protective phenotype observed in microbiota-intact CLA-treated mice. Survival rates were comparable between CLA and control groups, and although CLA-treated animals showed a trend toward lower bacterial burdens in the liver and spleen, the differences were not statistically significant (Figure 8B,C). Histopathological and immunohistochemical analyzes revealed similar degrees of epithelial damage and inflammatory infiltration in both groups (Figure 8D,E). Similarly, intestinal permeability assessed by FITC–dextran remained unchanged, and serum levels of TNF and IL-6 were only modestly reduced in CLA-treated mice (Figure 8F,G). Notably, despite the loss of overt protection, CLA treatment still enhanced CD8⁺ T-cell effector features within the ileal IEL compartment, including IFN-*γ* and granzyme B expression (Figure S7D), indicating that CLA can directly activate CD8⁺ T cells in a microbiota-independent manner. However, in the absence of an intact gut microbiota, this immune activation was insufficient to translate into effective mucosal protection *in vivo*. Together, these findings indicate that while CLA modulates CD8⁺ T cells responses, the presence of an intact intestinal microbiota is required for effective protection against *S.* Tm infection *in vivo*.

To further delineate the contribution of the microbiota, fecal microbiota transplantation (FMT) was performed using microbiota derived from CLA-treated or control donors into antibiotic-treated recipient mice (Figure 8H). Remarkably, FMT from CLA donors conferred significant protection against *S.* Tm infection, improving survival, reducing pathogen loads, and preserving mucosal integrity compared with control FMT (Figure 8I–L). Serum FITC–dextran permeability and proinflammatory cytokine levels were likewise reduced in CLA-FMT recipients (Figure 8M). In contrast, FMT from CLA-treated donors failed to confer full protection in CD8⁺ T-cell–depleted recipients, indicating that microbiota transfer alone is not sufficient to confer full protection in the absence of CD8⁺ T cells (Figure 8I–M). Collectively, these data support a model in which both CLA-associated microbiota changes and CD8⁺ T-cell responses participate in host protection against *S.* Tm infection.

## Discussion

**Figure 9.**
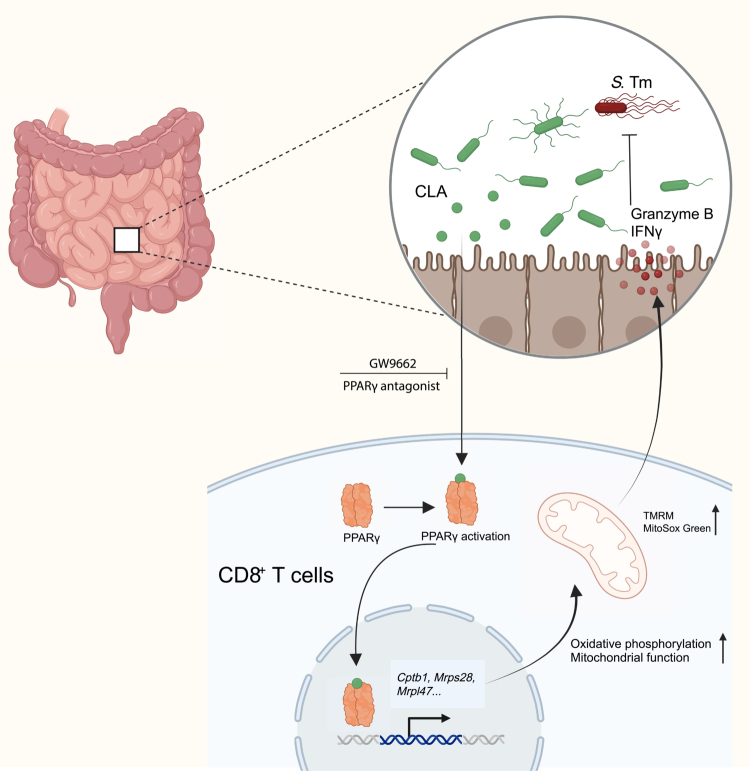
CLA enhances mucosal immune defense against *Salmonella* infection. Dietary CLA modulates intestinal immune homeostasis by reprogramming CD8⁺ T cells through PPARγ-mediated metabolic activation. CLA enhances mitochondrial biogenesis and oxidative phosphorylation, boosting IFN-*γ* and granzyme B production to strengthen mucosal defense against *Salmonella* infection.

In this study, we provide novel insights into the role of CLA in modulating intestinal immunity through metabolic reprogramming of intraepithelial lymphocytes. We demonstrate that CLA supplementation is associated with coordinated changes in gut microbial composition, metabolite profiles, and mucosal immune responses. Our results show that CLA enriches beneficial microbial taxa, modulates metabolite profiles, and activates immune cells, particularly CD8⁺ T cells, through PPARγ-mediated mitochondrial reprogramming. These findings provide compelling evidence that CLA not only influences microbial metabolism but also directly enhances immune responses via immune cell metabolic programming (Figure 9).

CLA, comprising positional and geometric isomers of linoleic acid—mainly *cis*-9, *trans*-11 and *trans*-10,*cis*-12 forms—has been widely recognized for its broad anti-inflammatory and metabolic benefits.^35^^,^^36^ Its anti-tumor and metabolic regulatory effects have been linked to modulation of the PI3K/Akt and p53 pathways, induction of caspase-dependent apoptosis, and activation of mitochondrial metabolism.^37^ Additionally, CLA ameliorated obesity-impaired mammary gland development through CD36 palmitoylation and downstream JNK–ERK signaling.^38^ CLA also promotes IL-35 expression in macrophages through Gαq/11–STAT1/4 signaling and suppresses systemic inflammation by reducing circulating TNF and IL-1β.^21^^,^^39^ In intestinal disease models, CLA alleviates DSS-induced colitis by reducing proinflammatory cytokines (TNF, and IL-6), enhancing epithelial tight junctions, and restoring microbiota composition.^40^ Notably, CLA supplementation in *Brachyspira hyodysenteriae*–infected pigs reduced mucosal injury and restored colonic PPARγ expression while maintaining CD4⁺ and CD8⁺ lymphocyte subsets.^41^ Our findings expand current understanding by revealing a previously unrecognized role of CLA in directly reprogramming intestinal CD8⁺ T cells toward a metabolically active and functionally enhanced state. Through the promotion of mitochondrial fitness and effector molecule expression, CLA fortifies mucosal immune defenses against enteric infection.

Intraepithelial lymphocytes, particularly CD8⁺ T cells, are key players in mucosal defense against *Salmonella* by recognizing and killing infected epithelial cells.^42^ It has been demonstrated that mucosal IFN-γ⁺/CD8⁺ T cells are essential for host protection against *Salmonella* infection.^43^ CD8⁺ T cells can recognize epitopes embedded within T3SS effector proteins, contributing to infection control and clearance through MHC-I–restricted antigen presentation.^44^^,^^45^ Indeed, CD8⁺ T cells targeting T3SS-derived antigens are capable of restricting *Salmonella* replication and dissemination.^46^ In the present study, we identified several distinct CD8⁺ T-cell subsets within the intestinal epithelium, including CD8αα⁺TCRαβ⁺/γδ⁺ and CD8αβ⁺TCRαβ⁺ effector and memory populations, based on marker gene expression such as *Gzmb*, *Trac*, *Trdc*, and *Tcf7*. This comprehensive characterization substantially expands our understanding of mucosal immune heterogeneity and its functional complexity. Furthermore, we found that CLA treatment primarily influenced the effector compartment, promoting a phenotypic shift from CD8αα⁺TCRαβ⁺/γδ⁺ toward CD8αβ⁺TCRαβ⁺ effector T cells. Although their overall abundance remained comparable between groups, CLA markedly enhanced oxidative phosphorylation and effector functions in both subsets. Whether these two IEL subsets exert distinct roles in mucosal immunity warrants further investigation.

CD4⁺CD8αα⁺ IELs possess both cytotoxic and regulatory functions and are critical for maintaining intestinal immune tolerance and anti-infective defense.^47^^,^^48^ In our study, CLA supplementation markedly increased the proportion of this subset, consistent with previous reports.^47^ Recent studies have shown that microbial metabolites, including indole derivatives from *Lactobacillus reuteri* and linoleic acid isomers produced by commensal bacteria, promote the accumulation of CD4⁺CD8αα⁺ IELs in the small intestine.^47^^,^^49^ Similarly, host-derived factors such as Apolipoprotein L9a/b have been identified as key protective effectors against *Salmonella*, as *Apol9a/b*-deficient mice exhibit elevated mortality, severe inflammation, and extensive bacterial dissemination.^50^ Beyond T cells, *Salmonella* infection reprograms multiple immune compartments,^51^ including CD62L⁺ monocytes that act as bottlenecks for bacterial dissemination.^52^ Additionally, *Salmonella* infection drives functional reprogramming of Vγ6⁺ Tγδ17 cells toward type 1 effector phenotypes via an AP-1 regulatory axis centered on JUNB and FOSL2, which modulates IL-17A production and maintains the balance between type 3 and type 1 mucosal immunity.^53^

Previous studies have established that CLA and its oxidized derivatives act as natural PPARγ ligands, directly binding to the receptor and inducing PPAR-responsive genes involved in fatty acid oxidation and apoptosis regulation.^54^ Notably, the *cis-9, trans-11* CLA isomer enhances PPARγ expression and activity across diverse cell types, including epithelial and immune cells.^55^ Previous evidence that the loss of PPARγ abrogates the anti-inflammatory effects of CLA in DSS- and CD4⁺ T cell–induced colitis models further reinforces the mechanistic dependence of CLA’s immunoregulatory actions on PPARγ signaling.^56^ In parallel, other lipid-derived metabolites, such as ursodeoxycholic acid, have been shown to activate PPARγ, increase fatty acid oxidation, and preserve tissue bioenergetics under stress,^57^ while gut microbiota-derived hydroxy-cholic acids promote intestinal type 3 immunity via FXR–WTAP–m6A–RORC signaling, offering additional protection against *Salmonella* infection.^58^ Consistent with these findings, we observed that CLA supplementation upregulated PPARγ target genes (*Mrps28*, *Elovl6*, *Mrpl47*, *Oxr1*, and *Immp2l*) in intestinal immune cells, promoting oxidative phosphorylation and mitochondrial activation. Importantly, pharmacological inhibition of PPARγ in mice attenuated these metabolic and protective phenotypes *in vivo*, indicating that PPARγ signaling contributes to CLA-associated immunometabolic remodeling. However, because PPARγ inhibition was achieved through systemic pharmacologic intervention, the present study does not establish the compartment-specific requirement of CD8^+^ T cells for PPARγ. Future studies employing conditional genetic models will be necessary to define the cell type–specific role of PPARγ in mediating CLA-associated host protection.

The gut microbiota is a fundamental determinant of resistance to enteric pathogens, acting through colonization resistance mechanisms that include nutrient competition, production of inhibitory metabolites, and maintenance of epithelial integrity.^59-61^ Disruption of microbial composition or diet compromises these defenses—for instance, loss of *Muribaculum intestinale*, a succinate-utilizing species, during fiber deprivation reduces propionate production and facilitates *S.* Tm overgrowth.^62^ Conversely, beneficial taxa such as *E. coli* 8178 limit *Salmonella* expansion through metabolic competition for galactose,^63^ whereas pasteurized *Akkermansia muciniphila* enhances GP2 expression in M cells and promotes infection independently of RANKL signaling.^64^ Engineered consortia such as SynComBac10 reinforce epithelial maturation and Th17 immunity, conferring resistance to *Salmonella*.^65^ The magnitude of protection varies among hosts with distinct microbial communities, correlating with enteropathy severity and neutrophil recruitment.^66^ Moreover, *Salmonella* infection reshapes the immune landscape by inducing hypoxia-responsive macrophages, depleting germinal-center B cells,^67^ and engaging in cross-kingdom interactions—*Candida albicans*, for example, releases extracellular arginine that activates the bacterial T3SS to enhance invasion.^68^ In parallel, bacterial effectors such as SopD and hydrogen sulfide production fine-tune inflammation and suppress commensal respiration, enabling *Salmonella* to overcome microbiota-mediated colonization resistance.^69^^,^^70^ Our data showed that CLA supplementation increased beneficial taxa such as *Duobesilla* and *Lactobacillus*, which may confer protection against enteric pathogens by stabilizing the gut microbial community and enhancing mucosal immunity.

Despite providing new insights into the role of CLA in shaping immunometabolic responses during enteric infection, several important questions remain. First, CD8⁺ T-cell depletion was achieved through systemic administration of anti-CD8α antibody, which does not permit compartment-specific attribution to ileal intraepithelial lymphocytes. Although our data indicate that CD8⁺ T cells contribute to the CLA-associated protective phenotype, we cannot formally distinguish between IEL-specific and systemic CD8-dependent effects. In addition, anti-CD8α–only infection controls were not included, and therefore we cannot exclude the possibility that systemic CD8 deficiency independently influences *Salmonella* susceptibility. Future studies employing tissue-specific conditional approaches will be necessary to determine the compartmental contribution of CD8⁺ T cells in this context. Second, CLA treatment promoted a phenotypic transition from CD8αα⁺TCRαβ⁺/γδ⁺ to CD8αβ⁺TCRαβ⁺ effector T cells. While transcriptomic analyzes indicate functional divergence among these subsets, our study does not establish which population is essential for CLA-mediated protection. Whether these subsets represent functionally distinct effectors or sequential differentiation states will require subset-specific loss-of-function studies in the future. Finally, although antibiotic treatment and fecal microbiota transplantation experiments demonstrate that microbiota integrity is required for the *in vivo* manifestation of CLA-associated protection against *Salmonella* infection, the present study does not establish a direct mechanistic linkage between microbiota remodeling and CD8⁺ T cells metabolic reprogramming. Broad-spectrum antibiotic treatment represents a global perturbation that may affect colonization resistance, inflammatory localization, and baseline immune tone simultaneously. Therefore, our findings do not distinguish whether the microbiota contributes through metabolic processing of CLA, provision of immune-modulatory signals, or microbiota-dependent colonization resistance independent of CD8⁺ T cells reprogramming. Future studies will be necessary to resolve these possibilities.

## Conclusion

In summary, our study demonstrates that dietary CLA enhances resistance to *Salmonella* infection and is associated with coordinated changes in microbial composition and mucosal immune responses. CLA supplementation enriches beneficial microbial taxa, restores metabolic homeostasis, and enhances CD8⁺ T cells function via PPARγ-mediated mitochondrial reprogramming. This integrated immunometabolic regulation not only limits pathogen colonization and inflammation but also reinforces mucosal integrity and tolerance. Together, these findings establish CLA as a key dietary lipid that bridges microbial metabolism and host immune fitness, highlighting its potential as a nutritional immunomodulator for preventing and mitigating enteric infections.

## Materials and methods

### Mice

Specific-pathogen-free (SPF) C57BL/6 mice (6–8 weeks old) were obtained from the Huachuang Sino (Jiangsu, China) and maintained under controlled temperature (22 ± 2 °C), humidity (55 ± 5%), and a 12 h light/dark cycle in the SPF animal facility of the Shanghai Veterinary Research Institute (SHVRI), Chinese Academy of Agricultural Sciences (CAAS). All mice had ad libitum access to a commercially available SPF-grade growth and breeding diet sterilized by Co60 irradiation (MD17111; Medicience, Jiangsu, China). Animals were acclimated for one week before experimentation. For each independent experiment, gender-matched mice were randomly assigned to experimental groups (*n* = 6–8 mice per group). All experiments were independently repeated at least twice to ensure reproducibility. At the start of dietary intervention, mice were 6–8 weeks of age and received CLA or vehicle control for 14 consecutive days. Mice were subsequently challenged with *S.* Tm and analyzed at predefined endpoints. For histological analysis, bacterial burden quantification, and immune phenotyping, mice were sacrificed at day 4 post-infection. For survival studies, mice were monitored daily for up to 21 d post-infection. For *in vivo* depletion of CD8⁺ T cells, mice were administered an anti-CD8α monoclonal antibody (clone 53-6.7, Biolegend) by intraperitoneal injection. Antibodies were injected at a dose of 250 μg per mouse on days −3 and −1 prior to *S.* Tm infection. For *in vivo* pharmacological inhibition of PPARγ, GW9662 (1 mg/kg; MCE, HY-16578) was dissolved in dimethyl sulfoxide and further diluted with sterile PBS prior to administration. Mice received intraperitoneal injections once daily for 14 consecutive days before *S.* Tm infection. The selected dosing regimen was based on previous studies demonstrating effective systemic inhibition of PPARγ signaling *in vivo* ^71^. All animal procedures were approved by the Institutional Animal Care and Use Committee (IACUC) of SHVRI (protocol approval number SV-20250425-G02) and conducted in accordance with the National Guidelines for the Care and Use of Laboratory Animals (China). All experiments were independently repeated at least twice to ensure reproducibility.

### *Salmonella enterica* serovar Typhimurium infection model

A streptomycin-resistant strain of *Salmonella* enterica serovar Typhimurium SL1344 used in this study was obtained from the lab stock. *S.* Tm was grown on LB agar plates supplemented with 50 mg/L streptomycin overnight at 37 °C. One colony was re-suspended in 10 ml LB broth and grown overnight at 37 °C with shaking at 200 rpm. Overnight bacterial culture (0.5 ml) was inoculated in 10 ml LB broth and further grown for another 4–6 h, followed by centrifugation at 9,000 × g for 5 min and re-suspension in ice-old sterile PBS for inoculation. For infection, mice were fasted for 4 h before oral gavage with 5 × 10^7^ colony-forming units (CFU) of *S.* Tm in 200 µL PBS. CLA-treated and control groups were infected following a 14 d pretreatment period with either CLA or PBS. Mice were euthanized at a predefined endpoint of day 4 post-infection, corresponding to an age of approximately 9–11 weeks, for tissue collection. Liver, spleen, and ileal contents were aseptically harvested to determine bacterial burdens. To assess early stages of *S.* Tm infection, Peyer’s patches and mesenteric lymph nodes were harvested at 6 h and 24 h post infection. Tissues were homogenized in sterile PBS, serially diluted, and plated on XLD agar containing 50 µg/mL streptomycin for enumeration of CFU.

### CLA preparation and oral administration

Conjugated linoleic acid (mixture of *cis*-9, *trans*-11 and *trans*-10, *cis*-12 isomers; Absin, China) was freshly prepared before each administration. Mice in the CLA group received 200 mg/kg body weight of CLA daily via oral gavage for 14 consecutive days, whereas control mice were gavaged with the same volume of PBS alone. The dosing regimen was selected based on previous studies demonstrating physiological relevance and mucosal immune modulation by dietary CLA.^19^^,^^72^ All treatments were performed at the same time of day to minimize circadian variation, and mice were monitored daily for body weight and general health status throughout the experimental period.

### Antibiotic treatment and fecal microbiota transplantation

To deplete the intestinal microbiota, mice (6–8 weeks old) were administered a broad-spectrum antibiotic cocktail containing ampicillin (1 g/L), neomycin (1 g/L), metronidazole (1 g/L), and vancomycin (0.5 g/L) in sterile drinking water for 14 consecutive days. Antibiotic-containing water was freshly prepared every two days and supplied ad libitum. For FMT experiments, fresh fecal pellets were collected from CLA-treated or control donor mice on day 14 of oral gavage, pooled within each group, and suspended in sterile PBS (100 mg/mL). The suspensions were homogenized and filtered through a 70-µm cell strainer to remove debris. Recipient mice (6–8 weeks old) were first subjected to the same 14 d antibiotic depletion protocol, then orally gavaged with 200 µL of freshly prepared fecal suspension three times within one week.

### Histopathology and immunohistochemistry

For histopathological analysis, ileal tissues were collected on day 4 post infection after *S.* Tm infection, gently flushed with cold PBS, and fixed in 4% paraformaldehyde overnight at 4 °C. Samples were dehydrated through graded ethanol, cleared in xylene, and embedded in paraffin. Sections of 4 µm thickness were prepared and stained with hematoxylin and eosin (H&E) using standard procedures. Histopathological evaluation was performed in a double-blinded manner based on villus and crypt morphology, epithelial integrity, and inflammatory-cell infiltration.

For immunohistochemical detection of MPO, paraffin sections were deparaffinized, rehydrated, and subjected to antigen retrieval in citrate buffer (pH 6.0) at 95 °C for 20 min. Endogenous peroxidase activity was quenched with 3% hydrogen peroxide for 15 min, followed by blocking with 5% bovine serum albumin for 30 min at room temperature. Sections were then incubated overnight at 4 °C with a rabbit anti-MPO primary antibody (1:200, Abcam), washed with PBS, and incubated with an HRP-conjugated goat anti-rabbit secondary antibody (1:500, Servicebio, China) for 1 h at room temperature. Signals were visualized using 3, 3′-diaminobenzidine, and nuclei were counterstained with hematoxylin. Images were captured using an Olympus BX53 microscope, and MPO-positive areas were quantified using ImageJ software from at least five randomly selected fields per section.

### Fluorescence In situ hybridization for *Salmonella* detection

Formalin-fixed, paraffin-embedded ileal sections (4 µm thick) were subjected to FISH for the detection of *Salmonella*. After deparaffinization in xylene and rehydration through graded ethanol, slides were incubated in 0.2 mol/L HCl for 10 min and treated with proteinase K (20 µg/mL, 37 °C, 15 min) to permeabilize bacterial cell walls. Sections were then hybridized overnight at 46 °C in hybridization buffer (20 mM Tris-HCl, 0.9 M NaCl, 0.01% SDS, 35% formamide) containing a *Salmonella*-specific 16S rRNA–targeted oligonucleotide probe (5'-UCCCGCUUAUUGAUAUGC-3'). After washing in buffer (20 mM Tris-HCl, 0.9 M NaCl, 0.01% SDS, 48 °C, 15 min), slides were counterstained with DAPI and mounted in antifade medium. Fluorescent signals were visualized using a confocal laser scanning microscope (Leica SP8, 100× oil objective). *Salmonella*-positive bacteria were identified as distinct red fluorescence signals within the intestinal lumen or epithelial layer, with DAPI marking host nuclei. *Salmonella*-positive fluorescent signals were quantified per field in ileal sections using ImageJ software. For each mouse, 3–5 randomly selected, non-overlapping fields were analyzed under identical imaging settings. Counts were performed in a blinded manner, and the mean value per mouse was used for statistical analysis.

### Measurement of intestinal permeability and cytokine levels

Intestinal barrier integrity was assessed using the FITC–dextran assay.^73^ Following an overnight fast, mice were orally gavaged with fluorescein isothiocyanate (FITC)–dextran (average molecular weight 4 kDa; MCE) at a dose of 0.6 mg/g body weight dissolved in sterile PBS.^74^ Four hours after gavage, blood was collected from the retro-orbital sinus into EDTA-coated tubes under isoflurane anesthesia and centrifuged at 3,000 × g for 10 min to obtain plasma. Plasma fluorescence was measured using a microplate reader (excitation 485 nm, emission 530 nm), and FITC–dextran concentrations were calculated from a standard curve generated with serial dilutions of FITC–dextran in mouse plasma. For cytokine quantification, whole blood was collected into serum-separation tubes and allowed to clot at room temperature for 2 h, followed by centrifugation at 3,000 × g. Serum levels of TNF and IL-6 were determined using mouse ELISA kits (Beijing 4 A Biotech, China) according to the manufacturer’s instructions.

### Isolation of intraepithelial lymphocytes and flow cytometry

IELs were isolated as previously described with minor modifications.^75^ Briefly, 10-cm ileal segments from CLA-treated and PBS-treated C57BL/6 mice were excised, Peyer’s patches were removed, and tissues were opened longitudinally and rinsed thoroughly with ice-cold PBS to remove luminal contents. The cleaned tissues were cut into 0.5–1 cm fragments and incubated in PBS containing 5 mM EDTA and 1 mM dithiothreitol at 37 °C for 30 min with gentle shaking to release epithelial-associated lymphocytes. The supernatant containing IELs was passed through a 70 μm cell strainer and centrifuged at 500 × g for 5 min. The cell pellet was resuspended in 40%/70% Percoll (GE Healthcare) and centrifuged at 600 × g for 20 min at room temperature without brake. IELs were collected from the interphase, washed, and resuspended in RPMI-1640 medium supplemented with 10% fetal bovine serum (FBS).

Single-cell suspensions were incubated with anti-CD16/32 (2.4G2, BD Biosciences) to block Fc receptors for 10 min at 4 °C, live and dead cells staining were used the fixable viability dye eFluor™ 506 (eBioscience), followed by staining with fluorochrome-conjugated antibodies against CD45 (30-F11), TCRβ (H57-597), CD4 (GK1.5), CD8α (53-6.7), and CD8β (YTS156.7.7) for 30 min at 4 °C in the dark. For intracellular cytokine detection, cells were stimulated with PMA (50 ng/mL) and ionomycin (1 μg/mL) in the presence of Brefeldin A (10 μg/mL) for 4 h, fixed and permeabilized using the Cytofix/Cytoperm™ Kit (BD Biosciences), and stained for IFN-*γ* (XMG1.2) and Granzyme B (QA16A02). For myeloid panel staining, CD45, CD11b (4AM1/70), F4/80 (4ABM8), Ly6C (4AHK1.4), Ly6G (1A8), CD11c (4AN418), and MHC-II (4ANIMR-4) antibodies were used the staining and the fluorescence’s were detected on NovoCyte 3130 flow cytometer (Agilent Technologies, USA). Data were analyzed using FlowJo software (BD Biosciences, USA).

### 16S rRNA gene sequencing and bioinformatic analyzes

Ileal luminal contents from CLA-treated and control mice were aseptically collected and immediately stored at –80 °C until DNA extraction. Microbial genomic DNA was isolated using the FastPure Stool DNA Isolation Kit (Magnetic bead) (MJYH, shanghai, China) following the manufacturer’s protocol. DNA integrity and concentration were assessed by 1% agarose gel electrophoresis and a NanoDrop 2000 spectrophotometer (Thermo Fisher Scientific, USA). The V3–V4 hypervariable regions of the bacterial 16S rRNA gene were amplified using primers 338F (5′-ACTCCTACGGGAGGCAGCAG-3′) and 806 R (5′-GGACTACHVGGGTWTCTAAT-3′) on a BIO-RAD T100 thermal cycler. Each 20 µL PCR reaction contained 4 µL 5 × FastPfu Buffer, 2 µL 2.5 mM dNTPs, 0.8 µL of each primer (5 µM), 0.4 µL FastPfu Polymerase, and approximately 10 ng template DNA. Cycling conditions were: initial denaturation at 95 °C for 3 min; 27 cycles of 95 °C for 30 s, 55 °C for 30 s, and 72 °C for 45 s; and a final extension at 72 °C for 10 min. PCR products were purified using the AxyPrep DNA Gel Extraction Kit (Axygen Biosciences, USA), quantified with a Qubit 4.0 fluorometer (Thermo Fisher Scientific), and then barcoded using primers carrying specific barcode sequences for each sample. The barcoded PCR products were pooled in equimolar concentrations and sequenced on an Illumina Nextseq 2000 PE300 (Illumina, USA).

After sequencing, the resulting data were demultiplexed using a custom Perl script to assign sequences to their respective samples. The sequences were then quality filtered with fastp (v0.19.6) and merged with FLASH (v1.2.11).^76^ Then the high-quality sequences were de-noised using DADA2 plugin in the Qiime2 (v2024.2) pipeline^77^ with recommended parameters, including a quality score truncation of 10 for both forward and reverse reads (trunc_len = 10) and a minimum sequence length filter of 100 (min_len = 100). DADA2 denoised sequences are usually called amplicon sequence variants (ASVs).^78^ Taxonomic classification was performed against the SILVA 138.2 reference database using a Naïve Bayes classifier.^79^ Alpha diversity indices (Shannon Index, which measures species diversity by accounting for both richness and evenness, and Pielou’s Evenness, which specifically evaluates the even distribution of species relative to the total richness) and beta diversity (Bray-Curtis dissimilarity) were calculated using the vegan R package (v2.7-1).^80^ Group differences in beta diversity were assessed using PERMANOVA (Permutational Multivariate Analysis of Variance).^81^ Differentially abundant taxa between groups were identified using the Wilcoxon rank-sum test at the genus level. Functional profiles predicted by Tax4Fun (v0.3.1) were further analyzed using linear discriminant analysis effect size (LEfSe) to identify KEGG pathways that differed between experimental groups. Pathways with an LDA score ≥ 2.0 and *P* < 0.05 were considered differentially represented and retained for downstream visualization. LEfSe results were visualized in R using the ggplot2 package (v3.5.2), with LDA scores used to represent pathway-level effect sizes.

### Metabolomic profiling of ileal luminal contents

Approximately 100 mg of ileal content per sample was transferred into a 2 mL centrifuge tube containing a 6 mm stainless-steel grinding bead and 800 µL of extraction solvent (methanol:water = 4:1, v/v) supplemented with internal standards (0.02 mg/mL L-2-chlorophenylalanine and others). Samples were homogenized for 6 min at –10 °C and 50 Hz using a Wonbio-96c tissue grinder (Wanbo Biotechnology, Shanghai, China), followed by ultrasonic extraction for 30 min at 5 °C and 40 kHz. Extracts were incubated at –20 °C for 30 min and centrifuged at 13,000 × g for 15 min at 4 °C. The resulting supernatants were transferred to autosampler vials for LC–MS/MS analysis. A pooled quality control (QC) sample was prepared by mixing equal aliquots from all samples and analyzed periodically (every 10 injections) to monitor analytical stability. LC–MS/MS analyzes were performed on a UHPLC–Orbitrap Exploris 240 system equipped with an ACQUITY HSS T3 column (100 × 2.1 mm, 1.8 µm; Waters, USA). The mobile phases consisted of (A) 0.1% formic acid in water and (B) acetonitrile, with a flow rate of 0.4 mL/min, column temperature of 40 °C, and injection volume of 5 µL. The mass spectrometer was operated in both positive and negative electrospray ionization (ESI) modes, with a source temperature of 400 °C, sheath gas at 40 arb, auxiliary gas at 10 arb, and ion-spray voltages of +3500 V and –2800 V. Data were acquired in data-dependent acquisition (DDA) mode over an m/z range of 70–1050.

Raw LC–MS data were processed using Progenesis QI (Waters Corporation, USA) for peak detection, alignment, and normalization. Metabolites were annotated by matching MS/MS spectra against the Human Metabolome Database, METLIN, and the Majorbio in-house database. Features with relative standard deviation > 30% in QC samples were removed before statistical analysis. Data were log₁₀-transformed and pareto-scaled. Differential metabolites between CLA-treated and control groups were identified using Student’s t-test (*p* < 0.05) and Variable Importance in the Projection (vip > 1). Pathway enrichment analysis of significantly altered metabolites was performed using the clusterProfiler package (v4.14.6) in R, with KEGG database annotations.

### Integrated 16S rRNA gene sequencing and metabolomics analysis

Integrated analysis of gut microbiota and metabolomics was performed to explore associations between microbial composition and CLA-related metabolites. Spearman correlation analysis was conducted to assess relationships between bacterial genera and metabolites. The top 10 bacterial genera were selected based on their mean relative abundance across all samples. CLA-related metabolites were identified from untargeted metabolomics data based on chemical annotation. Metabolites annotated as octadecadienoic or octadecatrienoic acid derivatives, including hydroxylated, dihydroxylated, oxo-, or nitro-substituted forms, and structurally related to CLA, were classified as CLA-related metabolites and retained for downstream correlation analyzes. Spearman’s rank correlation coefficients were calculated between selected bacterial genera and CLA-related metabolites using the *scipy.stats* package in Python (version 2.7.10). Correlation results were visualized as heatmaps, and statistical significance was indicated as *P* < 0.05 (*) and *P* < 0.01 (**).

### RNA sequencing and data analysis

Total RNA was extracted from 1 cm ileal tissues of CLA-treated and control mice using TRIzol™ Reagent (Invitrogen, USA) following the manufacturer’s instructions. RNA purity and concentration were determined with a NanoDrop 2000 spectrophotometer (Thermo Fisher Scientific, USA), and RNA integrity was assessed using the Agilent 5300 Fragment Analyzer (Agilent Technologies, USA). Samples with an RNA Quality Number (RQN) > 7.0 were used for library construction. RNA sequencing libraries were prepared using the Illumina® Stranded mRNA Prep, Ligation (Illumina, USA) kit according to the manufacturer’s protocol. Library size distribution and integrity were verified on the Agilent 5300 system, and concentrations were quantified using a Qubit 4.0 fluorometer (Thermo Fisher Scientific, USA). The libraries were then sequenced on an Illumina NovaSeq X Plus platform (Illumina, USA) to generate paired-end 150 bp reads.

Raw sequencing reads were processed for quality control using fastp (v0.19.6) to remove adapter sequences, low-quality reads, and reads containing poly-*N* stretches.^82^ Clean reads were aligned to the *Mus musculus* (GRCm39) reference genome using HISAT2 (v2.1.0),^83^ and transcript quantification was performed using RSEM (v1.3.3).^84^ Gene expression levels were normalized as transcripts per million. Differentially expressed genes (DEGs) between CLA-treated and control groups were identified using DESeq2 (v1.42.0),^85^ with thresholds of |log₂FoldChange| ≥ 1 and *p* < 0.05. Functional enrichment analysis of DEGs was conducted using GOatools (https://github.com/tanghaibao/GOatools) for GO terms and the Python scipy package (https://scipy.org/install/) for KEGG pathway enrichment. Enriched GO terms and KEGG pathways were visualized using ggplot2 (v3.5.2) in R.

### Single-cell RNA sequencing and data analysis

IELs were isolated from CLA- and PBS-treated mice infected with *Salmonella* on day 4 post-infection. Live CD45⁺ leukocytes were sorted using a BD FACSAria™ III cell sorter (BD Biosciences, USA) into RPMI-1640 medium supplemented with 10% FBS. Single-cell suspensions were loaded onto a Chromium Controller (10 × Genomics) using the Chromium Single Cell 3’ Library & Gel Bead Kit v3.1 according to the manufacturer’s instructions. Reverse transcription and cDNA amplification were performed following the 10 × Genomics user guide. Sequencing libraries were prepared using the Chromium Next GEM Single Cell 3′ Library Construction Kit and sequenced on an Illumina NovaSeq 6000 platform (paired-end 150 bp mode. Raw base call files were converted to FASTQ format using bcl2fastq (v2.20.0). Read alignment, barcode processing, and generation of the gene–cell count matrix were performed using Cell Ranger (v7.1.0) with the *Mus musculus* reference genome (GRCm39, Ensembl release 110). Downstream analyzes were conducted in Seurat (v5.1.0) in R. Low-quality cells were removed by excluding those with <200 or >6,000 detected genes or >10% mitochondrial gene content. Gene expression values were normalized and log-transformed using the *NormalizeData* and *ScaleData* functions. Highly variable genes were identified using *FindVariableFeatures*, and dimensional reduction was performed by PCA followed by Uniform Manifold Approximation and Projection (UMAP) for visualization. Cell clustering was conducted using *FindNeighbors* and *FindClusters* (Louvain algorithm) based on the first 30 principal components, with the default resolution set to 0.8 for clustering.

Major immune cell populations were annotated according to canonical marker genes and further verified using SingleR (v2.6.0) with the ImmGen mouse immune reference dataset (GSE109125). DEGs between CLA-treated and control groups were identified for each immune subset using *FindMarkers* (Wilcoxon rank-sum test) with thresholds of |log₂FC| ≥ 0.25 and adjusted *p* < 0.05. Functional enrichment of DEGs was analyzed using clusterProfiler (v4.14.6) for GO and KEGG pathways. For selected CD8⁺ intraepithelial lymphocyte subsets, cells were first subsetted from the integrated Seurat object based on refined cell-type annotations and treatment group (Control and CLA). Pseudo-bulk expression profiles were then generated using the Seurat function *AverageExpression*, which computes the mean normalized RNA expression (log-normalized data slot) across all cells within each subset and treatment condition. The resulting subset-level aggregated expression matrix was subsequently scaled on a gene-wise basis using Z-score transformation to facilitate relative comparison across genes. Heatmaps were visualized using the ComplexHeatmap package (v2.22.0) to depict subset-specific transcriptional programs across treatment groups. Intercellular communication networks were inferred using CellChat (v1.6.1) based on known ligand–receptor interactions. The total communication probability and pathway-specific information flow were calculated to identify signaling pathways modulated by CLA treatment.

### ATAC-seq and data analysis

Live CD45⁺ intraepithelial lymphocytes were sorted using fluorescence-activated cell sorting (FACS) with a BD FACSAria™ III (BD Biosciences, USA) from CLA-treated and PBS-treated mice infected with *Salmonella* on day 4 post-infection. Approximately 50,000 viable CD45⁺ nuclei per sample were subjected to the assay for transposase-accessible chromatin using sequencing (ATAC-seq) following the standard Tn5 transposase protocol.^86^ Transposed DNA fragments were purified with AMPure XP magnetic beads (Beckman Colter, USA), and library amplification cycles (average = 11) were determined by qPCR. Final libraries were quality-checked on an Agilent 5300 Fragment Analyzer using the D5000 ScreenTape assay and sequenced on an Illumina NovaSeq X Plus platform (Illumina, San Diego, USA) in paired-end 150 bp mode. Raw reads were processed for quality control using FastQC (v0.11.9) and trimmed with Trimmomatic (v0.39) to remove adapters and low-quality bases. Clean reads were aligned to the *Mus musculus* reference genome (GRCm39) using Bowtie2 (v2.4.5) with the parameters --very-sensitive -X 2000. PCR duplicates were removed with Picard MarkDuplicates, and open chromatin peaks were called using MACS2 (v2.1.1) (--extsize 150 --shift -75 --qvalue 0.05). Differentially accessible regions (DARs) were identified with DESeq2 (v1.42.0), and annotated using ChIPseeker (v1.32.0). GO and KEGG enrichment analyzes were performed using GOatools and the Python scipy package, respectively.

### Mitochondrial activity assessment

Naïve CD8⁺ T cells were isolated from the spleens of C57BL/6 mice according to the manufacturer’s instructions (Yeasen, China). Ninety-six–well plates were pre-coated with anti-CD3/CD28 antibodies (2 μg/mL) overnight at 4 °C to promote T cell activation. Cells (1 × 10⁵ per well) were cultured for 48 h in complete RPMI-1640 medium supplemented with different treatments, including CLA mixtures, individual isomers (*cis*-9, *trans*-11 and *trans*-10, *cis*-12), or the PPARγ inhibitor GW9662 (10 μM). After treatment, cells were stained with MitoTracker™ Green FM (200 nM) and TMRM (200 nM) (MCE) at 37 °C for 30 min to evaluate mitochondrial mass and membrane potential, respectively. Cell viability was assessed using DAPI (1 μg/mL) staining. Samples were acquired on a NovoCyte 3130 flow cytometer (Agilent Technologies, USA), and data were analyzed using FlowJo v10.8 (BD Biosciences, USA).

### Quantitative real-time PCR

Total RNA was extracted from purified CD8⁺ T cells and 1-cm distal ileal tissues using TRIzol™ Reagent (Invitrogen, USA) according to the manufacturer’s protocol. RNA concentration and purity were measured with a NanoDrop 2000 spectrophotometer (Thermo Fisher Scientific, USA). One microgram of total RNA was reverse-transcribed into cDNA using the PrimeScript™ RT reagent kit with gDNA Eraser (Takara, Japan). Quantitative PCR was performed on a Bio-Rad CFX96 real-time PCR system using SYBR® Green Master Mix (Takara, Japan). Each 20 μL reaction contained 10 μL of 2 × SYBR Green Mix, 0.4 μL of each primer (10 μM), 2 μL of cDNA template, and 7.2 μL of nuclease-free water. The thermal cycling conditions were as follows: 95 °C for 30 s, followed by 40 cycles of 95 °C for 5 s and 60 °C for 30 s. Relative mRNA expression levels of *Cpt1b*, *Mrps28*, *Mrpl47*, and *PPARγ* with primers were normalized to *β-actin* and calculated using the 2^–ΔΔCt method. The primer sequences used were as follows:

*Cpt1b* forward (F) 5′-ATCTTGGTGGCATGGCTGGT-3′, reverse (R) 5′-GGGACTGGTCGATTGCATCC-3′; *Mrpl47* F 5′-GGTGCTTGGAGAAGGGACATCT-3′, R 5′-GCGATCCACATAAGGCATTGCG-3′; *Mrps28* F 5′-GGCGCATCTTCCATATCGTGGA-3′, R 5′-CCAGGAACCTAGATGTCAGCTC-3′; *PPARγ* F CAGGCTTGCTGAACGTGAAG, *PPARγ* R GGAGCACCTTGGCGAACA; *β-actin* F 5′-CCCAGGCATTGCTGACAGG-3′, R 5′-TGGAAGGTGGACAGTGAGGC-3′.

### Assessment of energy production

Mitochondrial respiration and glycolytic activity were assessed in FACS-sorted CD8⁺ T cells using the Seahorse XF96 Extracellular Flux Analyzer (Agilent Technologies, USA) according to the manufacturer’s protocol. Approximately 5 × 10⁵ viable CD8⁺ T cells per well were seeded into poly-L-lysine–coated XF96 microplates (20 μg/mL) and centrifuged briefly to promote cell adherence. Cells were then incubated in non-buffered Seahorse assay medium (Agilent) in a non-CO₂ incubator at 37 °C for 30 min to equilibrate before measurement. For mitochondrial function, the XF Cell Mito Stress Test Kit (Agilent) was used to measure the OCR. After baseline recordings, cells were sequentially injected with 1 μM oligomycin, 1.5 μM FCCP, and 1 μM rotenone/antimycin A to assess basal respiration, ATP production, maximal respiration, and spare respiratory capacity. For glycolytic profiling, the XF Glycolysis Stress Test Kit (Agilent) was used. Cells were first incubated in glucose-free medium to establish baseline ECAR readings, followed by sequential injections of 10 mM glucose, 1 μM oligomycin, and 50 mM 2-deoxy-D-glucose (2-DG) to determine glycolysis, glycolytic capacity, and glycolytic reserve. All measurements were performed in triplicate. Data were analyzed using the Seahorse Wave software (Agilent Technologies).

### Statistical analysis

All data were analyzed using GraphPad Prism (version 10.0) and R software (version 4.3.2). Results are expressed as mean ± SEM. For comparison between two groups, statistical significance was determined using an unpaired two-tailed Student’s t-test or a Wilcoxon rank-sum test. For multiple group comparisons, one-way ANOVA followed by Tukey’s post hoc test was applied. Survival curves were analyzed using the log-rank (Mantel–Cox) test. Correlations between bacterial taxa and metabolites were computed using Spearman’s rank correlation. Single-cell RNA-seq data were processed in Seurat (v5.0), and DEGs were identified using the Wilcoxon test with Benjamini–Hochberg FDR correction for multiple comparisons. For ATAC-seq, DARs were identified were identified using DESeq2, with *p* values adjusted for multiple testing by the Benjamini–Hochberg FDR. Regions with FDR < 0.05 and |log₂ fold change| > 1 were considered significant. All experiments were independently replicated at least twice. Statistical significance is denoted as *p < 0.05, **p < 0.01, ***p < 0.001, ****p < 0.0001.

## Supplementary Material

Figure S2.pdf Figure S2.pdf

Figure S4.pdf Figure S4.pdf

Figure S1.pdf Figure S1.pdf

Figure S3.pdf Figure S3.pdf

Figure S7_1.pdf Figure S7_1.pdf

Figure S6.pdf Figure S6.pdf

Figure S5_1.pdf Figure S5_1.pdf

Supplementary material Supplementary material

## Data Availability

The 16S rRNA gene sequencing, bulk RNA-seq, and single-cell RNA-seq datasets generated in this study have been deposited in the NCBI Sequence Read Archive under BioProject accession number PRJNA1356503.
