# Supplementary figures and images for: Dietary conjugated linoleic acid enhances resistance to Salmonella infection by promoting PPARγ-mediated metabolic reprogramming and effector function in CD8⁺ T cells
Source: Gut Microbes. 2026 Apr 10;18(1):2657625. doi: 10.1080/19490976.2026.2657625 (PMC13078244; doi:10.1080/19490976.2026.2657625)

Figure S2

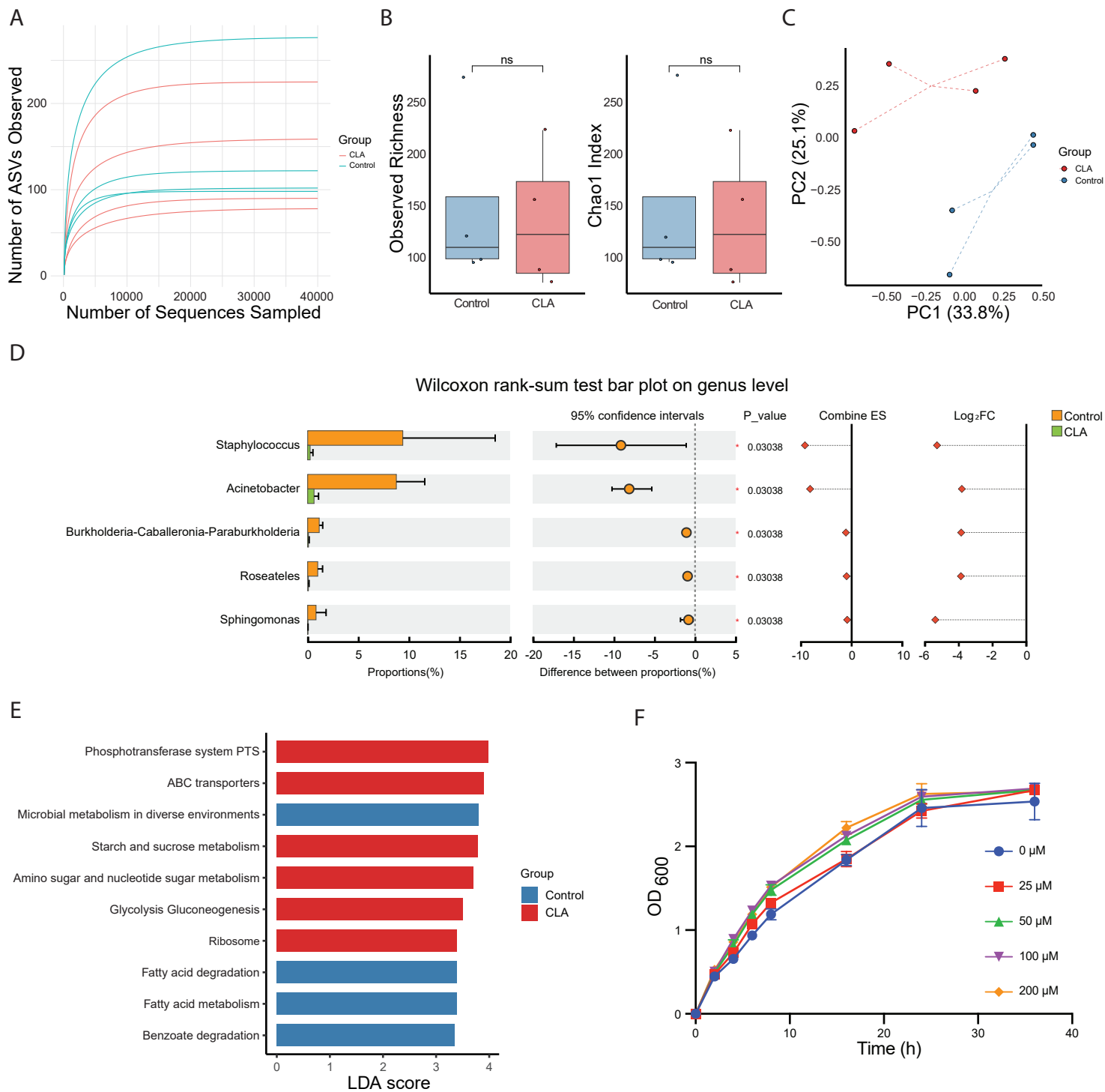

Supplement: Figure S2.pdf [file KGMI_A_2657625_SM1466.pdf]

Figure S4

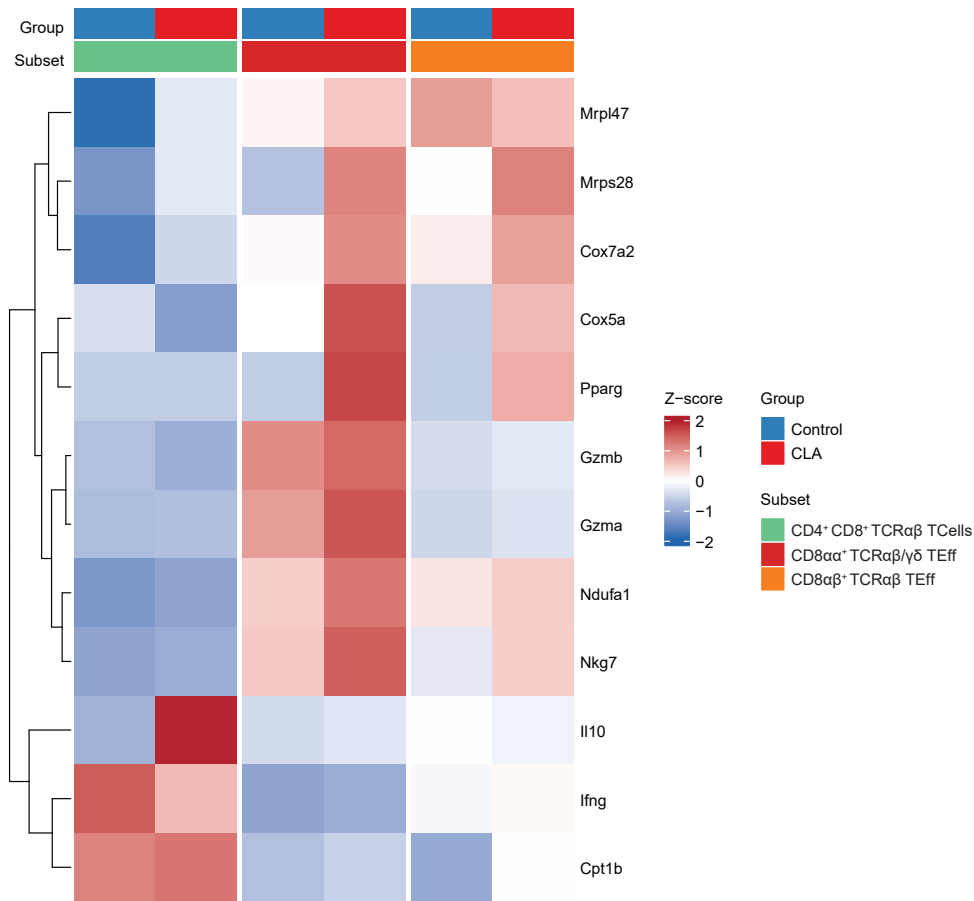

Supplement: Figure S4.pdf [file KGMI_A_2657625_SM1460.pdf]

Figure S1

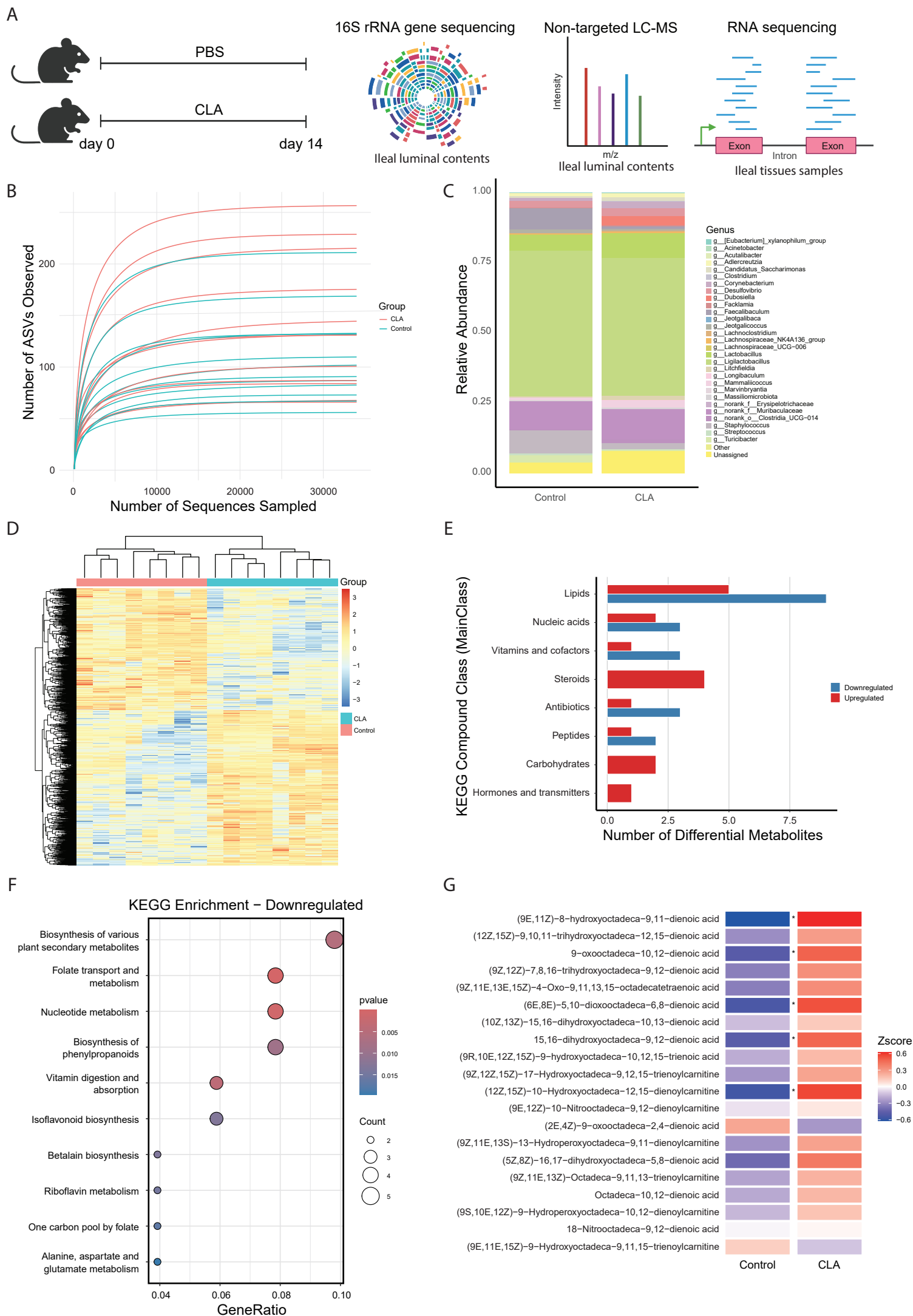

Supplement: Figure S1.pdf [file KGMI_A_2657625_SM1461.pdf]

A

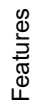

B

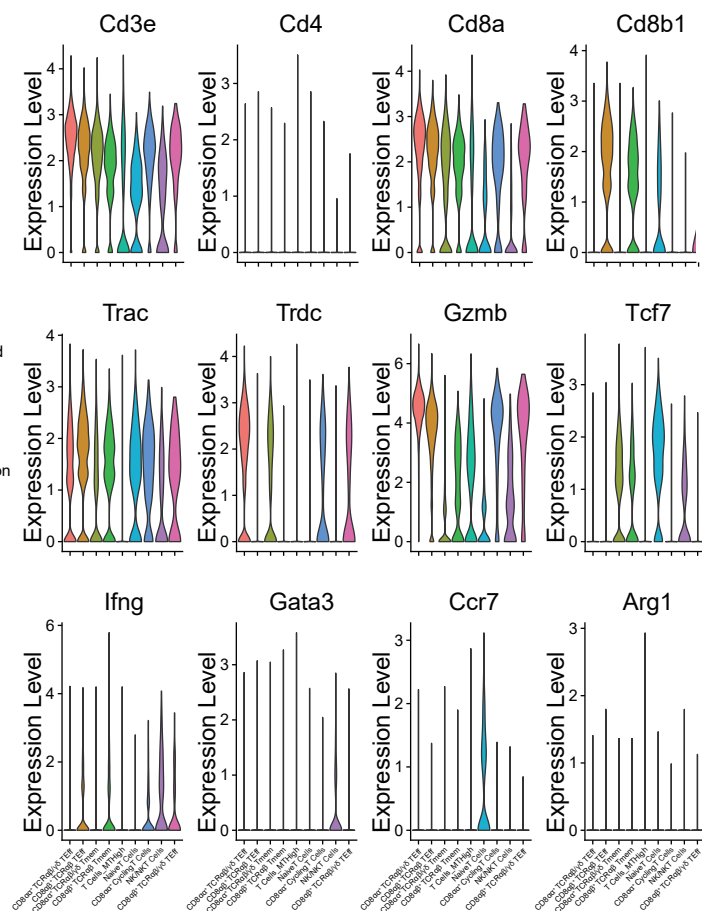

C

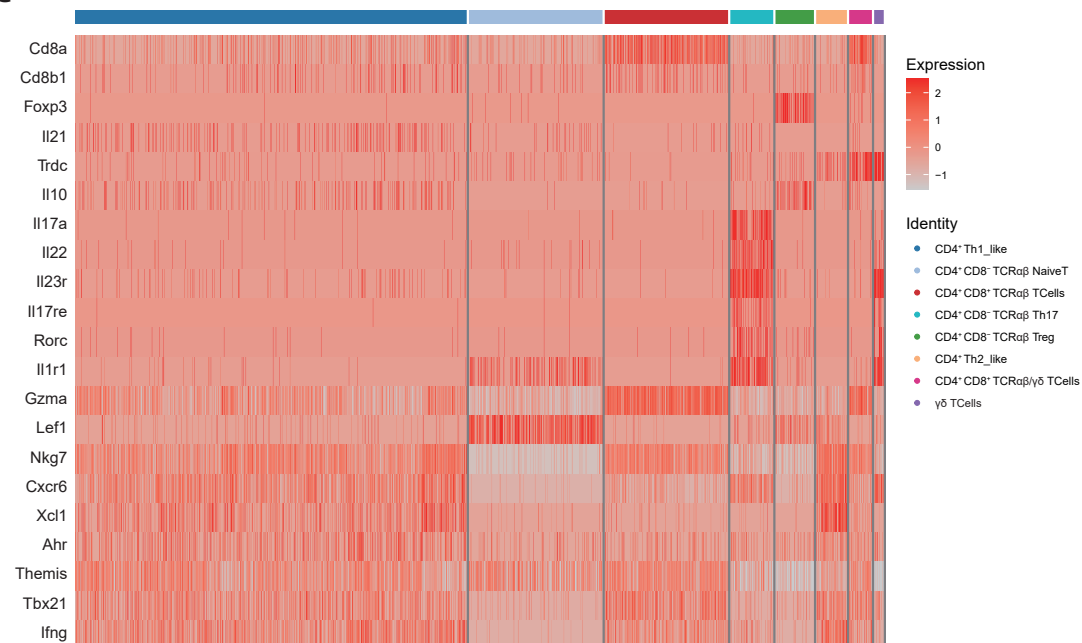

D

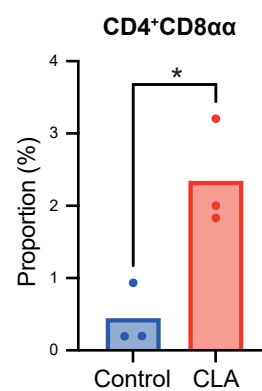

Supplement: Figure S3.pdf [file KGMI_A_2657625_SM1462.pdf]

Figure S7

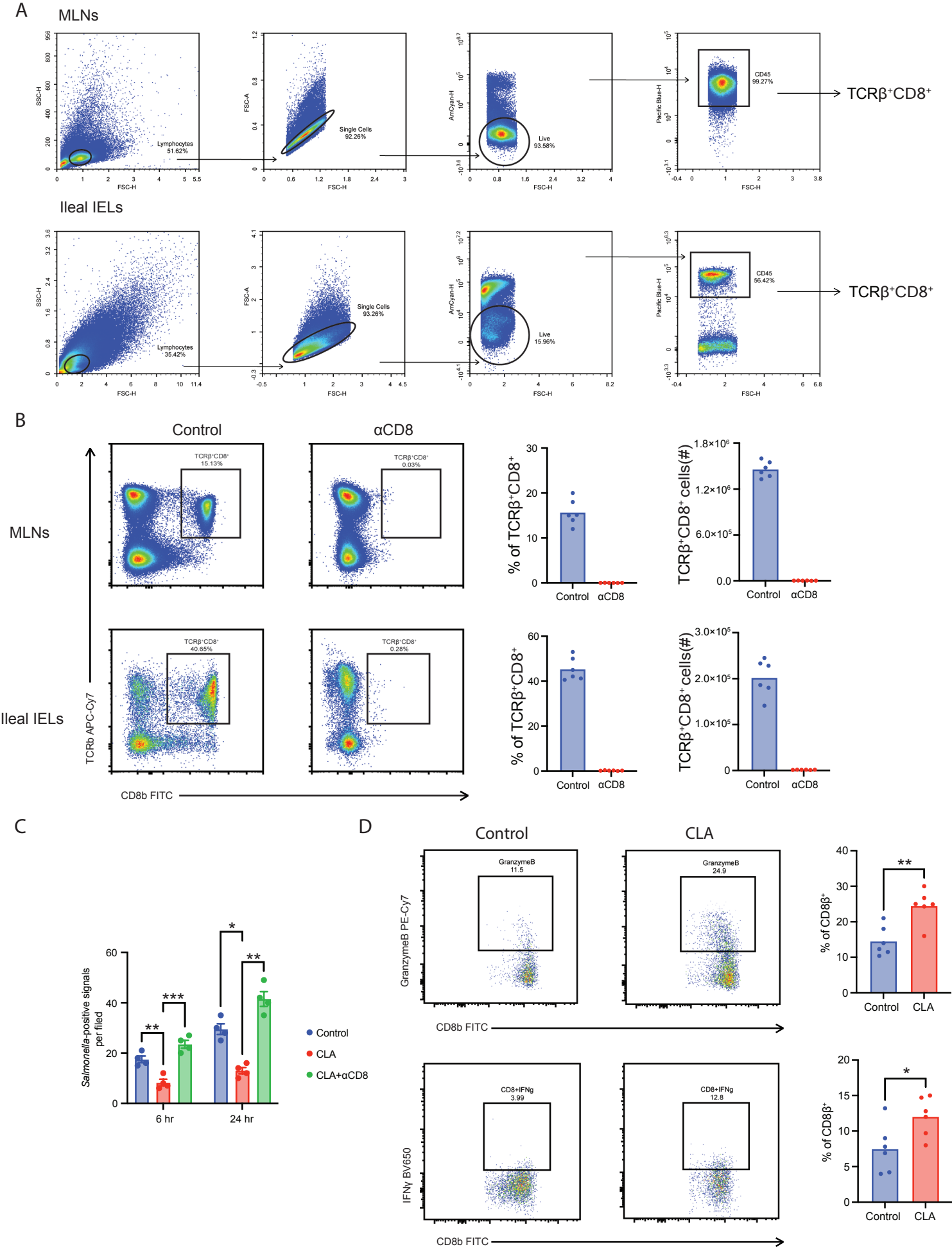

Supplement: Figure S7_1.pdf [file KGMI_A_2657625_SM1463.pdf]

Figure S6

A

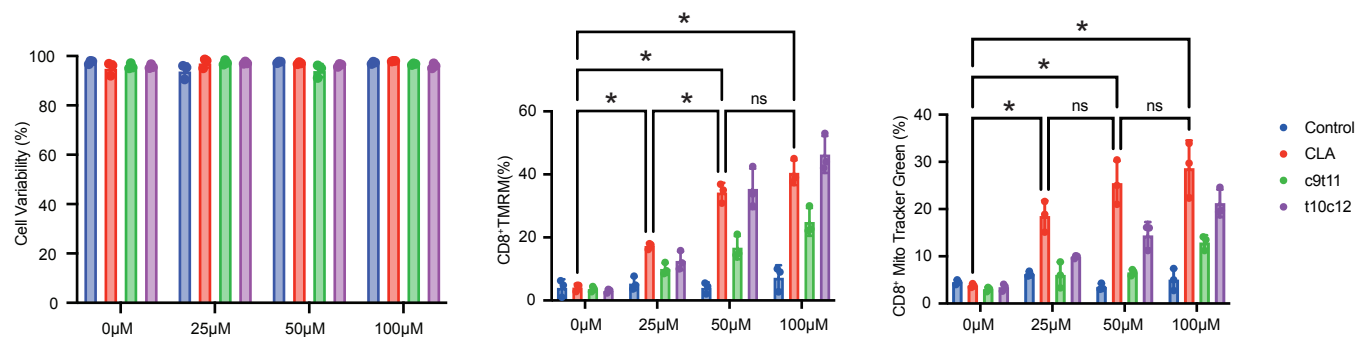

B

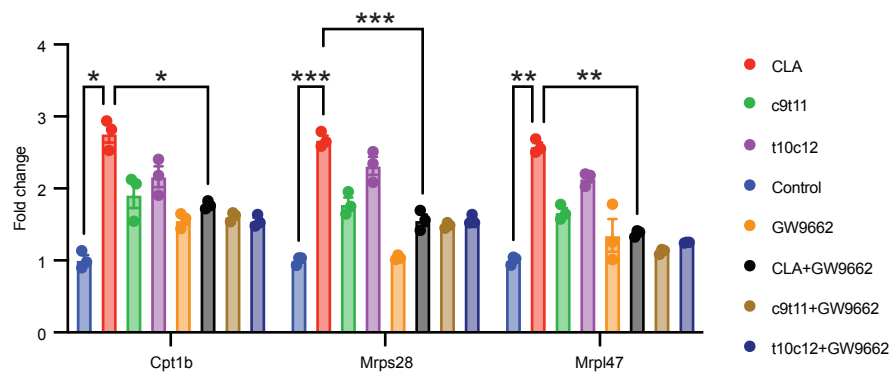

C

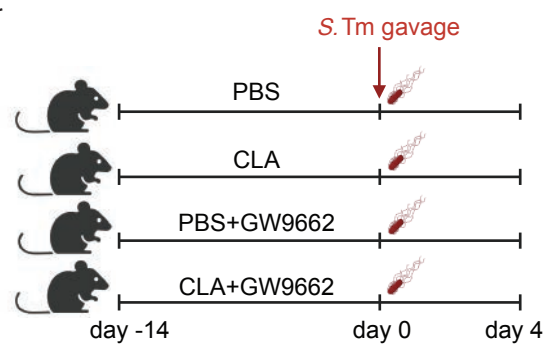

D

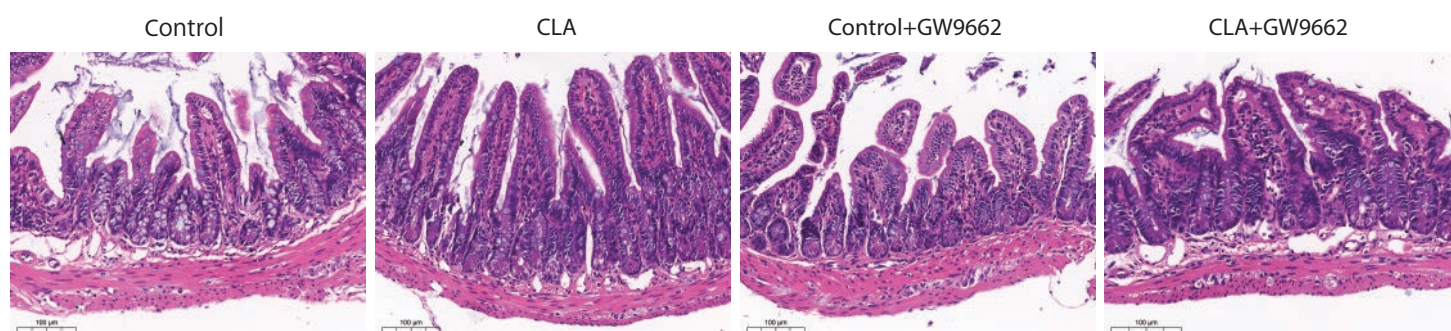

E

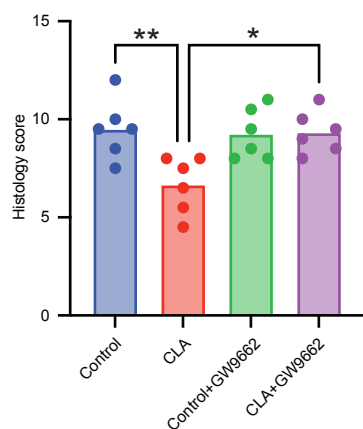

F

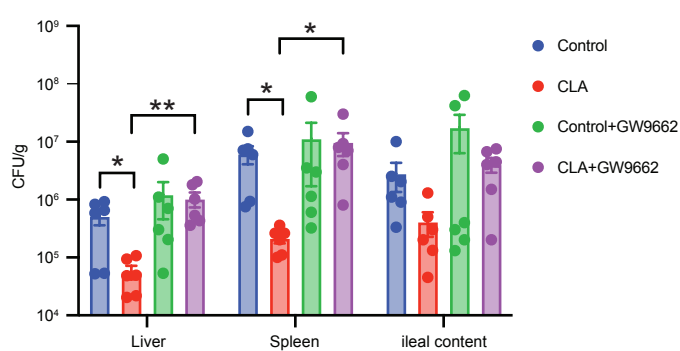

G

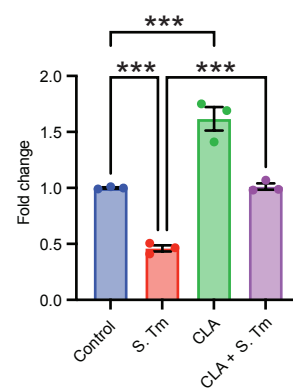

Supplement: Figure S6.pdf [file KGMI_A_2657625_SM1464.pdf]

Figure S5

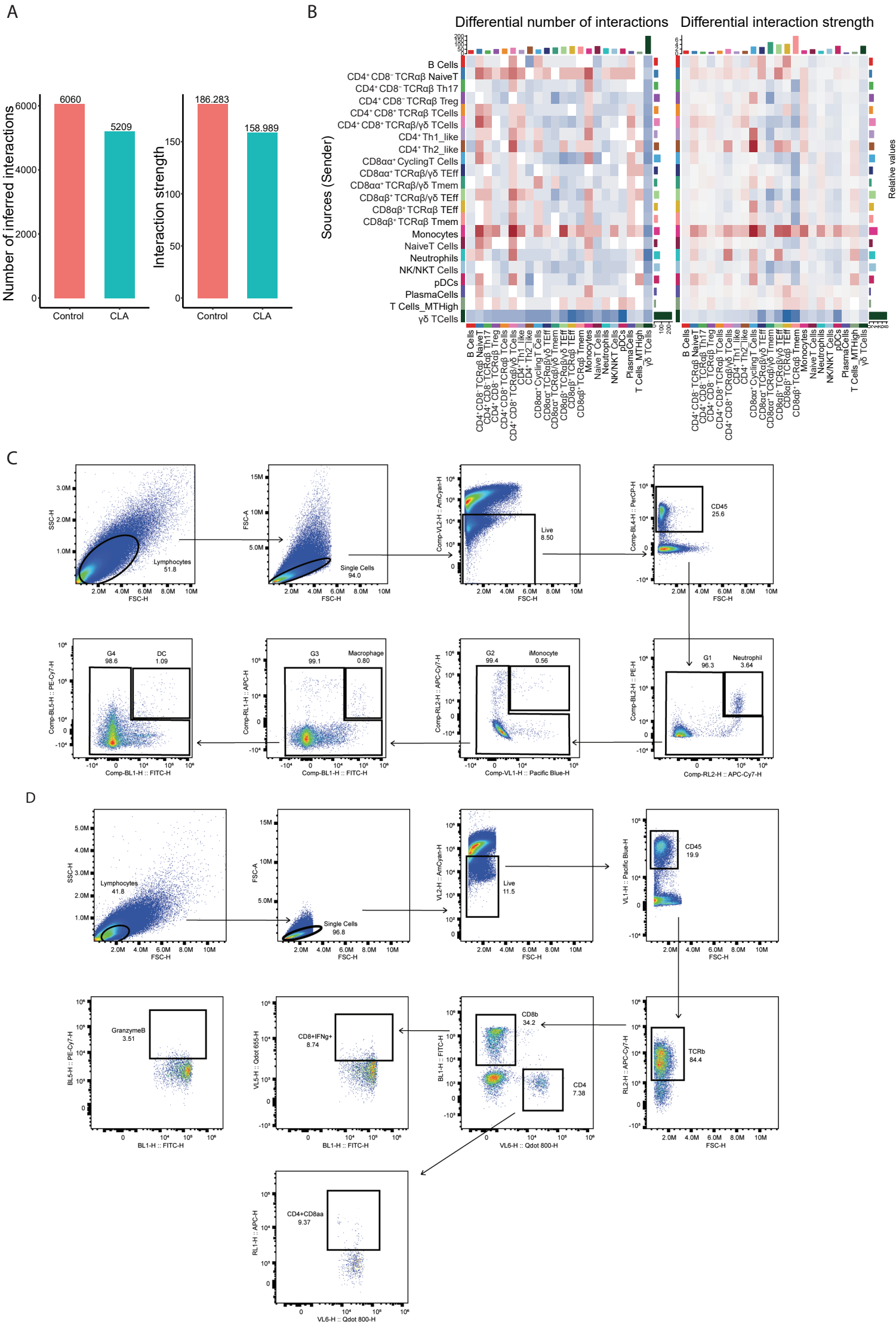

Supplement: Figure S5_1.pdf [file KGMI_A_2657625_SM1465.pdf]
