# Supplementary material for: Dietary conjugated linoleic acid enhances resistance to Salmonella infection by promoting PPARγ-mediated metabolic reprogramming and effector function in CD8⁺ T cells
Source: Gut Microbes. 2026 Apr 10;18(1):2657625. doi: 10.1080/19490976.2026.2657625 (PMC13078244; doi:10.1080/19490976.2026.2657625)
Supplement: Supplementary material [file KGMI_A_2657625_SM8266.docx]

**Supplementary information**

**Figure S1.** **CLA supplementation alters gut microbial, metabolic, and mucosal immune landscapes under homeostatic conditions.** (A) Experimental workflow of CLA supplementation followed by 16S rRNA gene sequencing, untargeted LC–MS metabolomics, and RNA sequencing. (B) Rarefaction curves showing sequencing depth and species richness across groups. (C) Taxonomic composition of ileal microbiota at the genus level in CLA-treated and control mice. (D) Hierarchical clustering heatmap of significantly altered metabolites. (E) KEGG compound classification of up- and downregulated metabolites. (F) KEGG enrichment analysis of downregulated metabolic pathways in CLA-treated mice. (G) Heatmap of CLA-derived oxylipins and acylcarnitines in ileal contents.

**Figure S2. CLA modulates intestinal microbial composition following *Salmonella* infection.** (A) Rarefaction curves showing sequencing depth and microbial richness across groups. (B) Alpha diversity indices of ileal microbiota in CLA-treated and control mice. (C) PCA based on Hellinger-transformed ASV tables. (D) Wilcoxon rank-sum analysis identifying significantly altered bacterial genera between groups. (E) Top 10 differential KEGG pathways identified by LEfSe analysis. (F) Growth curves of S. Tm cultured with different concentrations of CLA.

**Figure S3. Single-cell transcriptomic profiling of intraepithelial CD45⁺ immune cells.**
(A) Dot plot displaying the top DEGs among ileal intraepithelial CD45⁺ immune cell populations. Color intensity indicates the average expression level of each gene, while dot size represents the percentage of cells expressing the gene within each cluster. (B) Violin plots showing the expression distribution of selected marker genes among CD8⁺ T cells, highlighting key lineage and activation signatures. (C) Heatmap illustrating representative marker gene expression across eight transcriptionally distinct CD4⁺ T cells subpopulations. (D) Statistical comparison of CD4⁺CD8αα⁺ effector-like T cells between CLA-treated and control mice. Statistical significance was determined by unpaired Student’s t-test, **p* < 0.05.

**Figure S4.** Heatmap showing pseudo-bulk–averaged expression of representative immunoregulatory, cytotoxic, and mitochondrial/metabolic genes among CD4⁺CD8αα⁺ T cells, CD8αα⁺TCRαβ⁺/γδ⁺ effector T cells, and CD8αβ⁺TCRαβ⁺ effector T cells from control and CLA-treated mice infected with *Salmonella*.

**Figure S5.** **Flow cytometric validation and intercellular communication analysis.** (A, B) CLA treatment decreased both the total number and overall strength of predicted cell–cell interactions within the intestinal immune compartment. (C) Representative flow cytometry gating strategy for myeloid cell subsets shown in Figure 4D–F. (D) Gating strategy for intraepithelial T-cell subsets corresponding to Figure 4G–J.

**Figure S6. CLA enhances CD8⁺ T cells mitochondrial function and mucosal protection through PPARγ activation.** (A) Cell viability and mitochondrial parameters of CD8⁺ T cells treated with increasing concentrations (0–100 µM) of CLA (mixture of c9t11 and t10c12), c9t11, or t10c12 for 24 h. Mitochondrial membrane potential (TMRM) and mitochondrial mass (MitoTracker Green) were quantified by flow cytometry. (B) Relative mRNA expression of *Cpt1b*, *Mrps28*, and *Mrpl47* in CD8⁺ T cells following treatment with CLA isomers with or without the PPARγ antagonist GW9662 (10 µM). Data are normalized to *β-actin*. (C) Experimental design. (D) Representative H&E staining of ileal sections from control and CLA-treated mice, with or without GW9662 administration. (E) Quantification of histological inflammation scores (left) and (F) bacterial burdens (CFU/g) in liver, spleen, and ileal contents (right) from mice of the indicated groups after *S.* Tm infection. (G) Relative PPARγ mRNA expression in ileal intraepithelial lymphocytes under the indicated treatment conditions. c9t11 (cis-9,trans-11 CLA), t10c12 (trans-10,cis-12 CLA). Data are presented as mean ± SEM; **p* < 0.05, ***p* < 0.01, ****p* < 0.001, *****p* < 0.0001. Results are representative of at least two independent experiments.

**Figure S7.** (A) Representative sequential gating strategy for flow cytometric analysis of CD8⁺ T cells. Total acquired events were first gated based on FSC/SSC to identify lymphocytes, followed by singlet discrimination (FSC-A vs FSC-H), live/dead exclusion, and CD45⁺ leukocyte gating. (B) Representative flow cytometry plots showing TCRβ⁺CD8⁺ T cells gating from CD45⁺ T cells in mesenteric lymph nodes (MLNs) and ileal intraepithelial lymphocytes from control and anti-CD8α–treated mice. Quantification of both frequencies and absolute cell numbers is shown to assess depletion efficiency. (C) Quantification of *Salmonella* invasion in ileal sections at 6 h and 24 h post infection. (D) Representative intracellular staining and proportion of granzyme B⁺ and IFN-γ⁺ CD8⁺ T cells in antibiotic treated mice. Data are presented as mean ± SEM; **p* < 0.05, ***p* < 0.01, ****p* < 0.001, *****p* < 0.0001. Results are representative of at least two independent experiments.
